# Estimating cetacean population trends from static acoustic monitoring data using Paired Year Ratio Assessment (PYRA)

**DOI:** 10.1371/journal.pone.0264289

**Published:** 2022-03-17

**Authors:** Eric P. M. Grist, Trevelyan J. McKinley, Saptarshi Das, Tom Tregenza, Aileen Jeffries, Nicholas Tregenza

**Affiliations:** 1 Chelonia Ltd., Mousehole, Cornwall, United Kingdom; 2 College of Engineering, Mathematics and Physical Sciences, University of Exeter, Exeter, United Kingdom; 3 College of Medicine and Health, University of Exeter, Exeter, United Kingdom; 4 Institute for Data Science and Artificial Intelligence, University of Exeter, Exeter, United Kingdom; 5 Centre for Ecology & Conservation, School of Biosciences, University of Exeter, Penryn, United Kingdom; 6 Harbor Porpoise Project, Anacortes, Washington, United States of America; Hanyang University, REPUBLIC OF KOREA

## Abstract

The cetacean conservationist is often faced with evaluating population trends from abundance data that are either sparse or recorded at different times in different years. The presence of diel or seasonal patterns in the data together with unplanned gaps is often problematic. Such data are typical of those obtained from static acoustic monitoring. We present a simple and transparent non-parametric trend evaluation method, ‘Paired Year Ratio Assessment (PYRA)’ that uses only whole days of data wherever they are present in each of successive pairs of periods of 365 days. We provide a quantitative comparison of the performance of PYRA with traditional generalised additive models (GAMS) and nonparametric randomisation tests that require a greater level of skill and experience for both application and interpretation. We conclude that PYRA is a powerful tool, particularly in the context of identifying population trends which is often the main aim of conservation-targeted acoustic monitoring.

## Introduction

Assessing trends in populations is crucial to their conservation. Static acoustic monitoring of animal vocalisations has become increasingly useful for this purpose because it has the potential for long periods of monitoring and can deliver large sets of data at relatively lower cost than line transect survey methods [1–5]. The estimation of trends in the size and distribution of populations is distinct from estimating changes in absolute population size, and may be possible without sampling of the whole range of the species and with fewer observational data [6–8].

Here we address the problem of estimating population trends from data obtained by static acoustic monitoring of cetaceans. Such data may have been recorded at different times in different years [7,9–11] and may have other limitations. We do not address the question of site representativeness, but rather focus on methods for determining trends in data from one or more fixed sites even when there may be:

- strong diel and seasonal patterns of habitat use
- variable, unintended data gaps
- large variations in detection rates among sites
- constraints on time available to process data
- limited access to sophisticated statistical expertise
- a need to communicate with statistically non-expert readers

A trend is a smooth long term change or change in average tendency over a period of time [12–14]. We focus on the trend over the whole time span of the data set. Although sophisticated statistical approaches for estimating trends such as generalized additive modelling (GAM) or Bayesian methods can be employed, they do so at the cost of importing additional complexity requiring concomitant user skills for statistical inference [11,15,16]. Here we propose a simple and transparent non-parametric trend evaluation approach based on the changes over successive pairs of periods of 365 days. By ‘transparent’ we mean an approach in which errors or the failure of the method can be readily anticipated by users. This contrasts with more complex approaches, where it can be difficult for users and readers to evaluate whether the fit of a model to the data justifies the conclusions that can be drawn when all the assumptions of the model are met.

Cetaceans, like many highly mobile animals, often show strong diel and seasonal patterns of habitat use. These may confound assessment of longer trends, particularly when data have gaps in varying parts of days or years. To manage this issue our approach applies two rules: (1) only whole days of logging are used, and (2) comparisons across years use only those days that are ‘paired’ i.e., the days for which the same position in both years were both fully logged. This removes the need to estimate and manage diel and seasonal patterns. The extra day in leap years is omitted. In each year in the pair the detections on paired days are summed. Pairs of 365-day data windows can be moved through the time sequence in 1-day steps, giving a new pair of detection totals each time, and a ratio between them is to taken to provide an estimate of the change between the two points at the point in time midway between them.

We refer to this approach as Paired Year Ratio Assessment (PYRA) and compare this with trend estimation using randomisation tests and GAMs and express the method in non-technical terms as well as formal mathematical terminology.

Our aims are to:

1. Describe PYRA and compare its performance in case studies with other methods.
2. Identify key practical constraints.

## Methods

Among the more complex statistical approaches are some where the exact parametric form of the model is asserted, such as within a Bayesian framework [2,7] or others such as a GAM where it is not [11,17]. Here we compare the performance and robustness of PYRA as a descriptive statistical tool for trend estimation to results obtained with a GAM and with nonparametric randomisation trend testing [12].

### Synthetic data sets

We generated synthetic data sets with known, exact, intra-, and inter-annual trends in detections, plus added noise and a pattern of gaps that drifts through successive years. Our data exemplify the problem of gaps that by chance (or design in this case), over time, may show some correlation with the seasonal pattern. The potential impact was explored by considering two extreme case scenarios. In each, daily clicks fluctuations are specified by a Normal distribution.

#### Scenario 1

**Fig 1:**
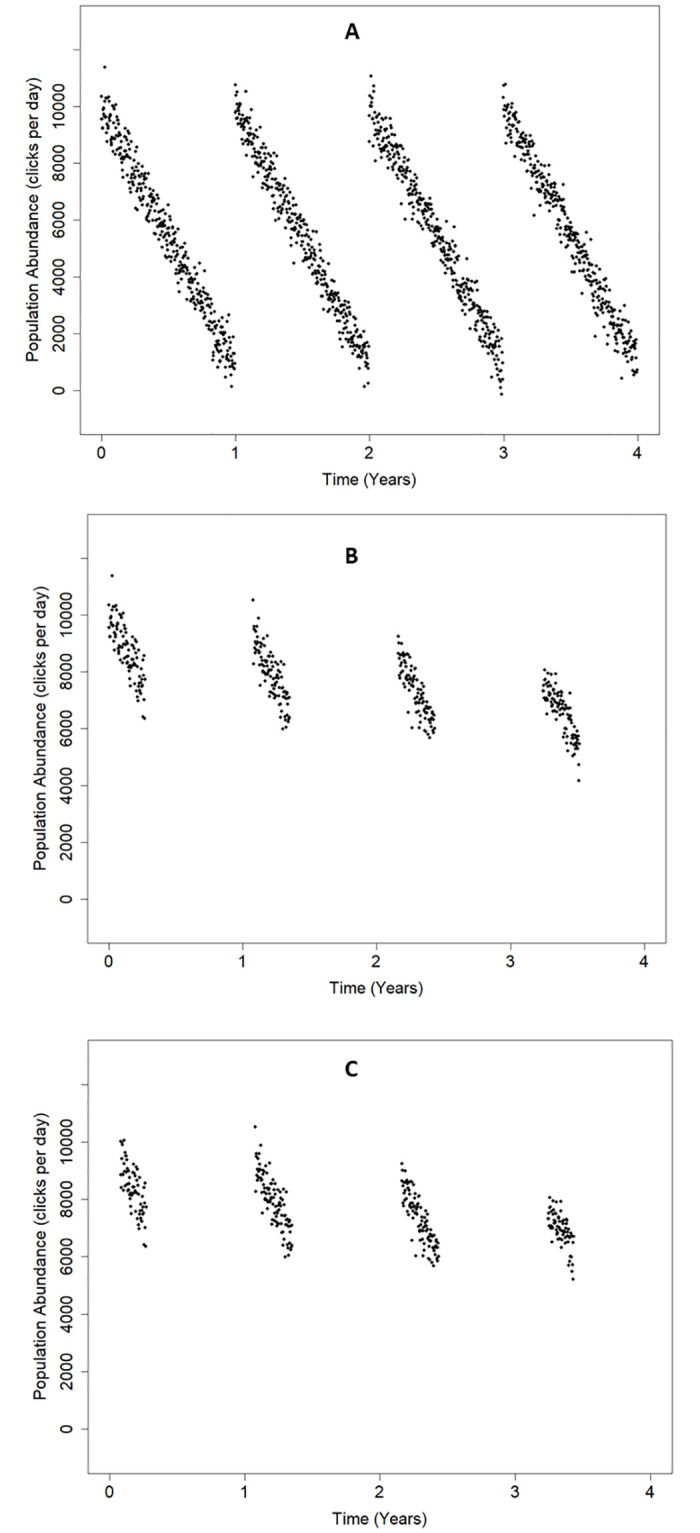
Scenario 1: ‘Seasonal’ detection pattern with no long-term trend. (A) *complete data*, (B) *incomplete data* and (C) *paired data*. The downward trend visible in (B) and (C) is a spurious outcome of the incomplete sampling which illustrates the type of problem that population trend estimation from incomplete data needs to address.

The population drops linearly within each year from a fixed mean value at the start of the year to a lower fixed mean value of at the year end, as shown in Fig 1. There is no downward trend across years.

#### Scenario 2

**Fig 2:**
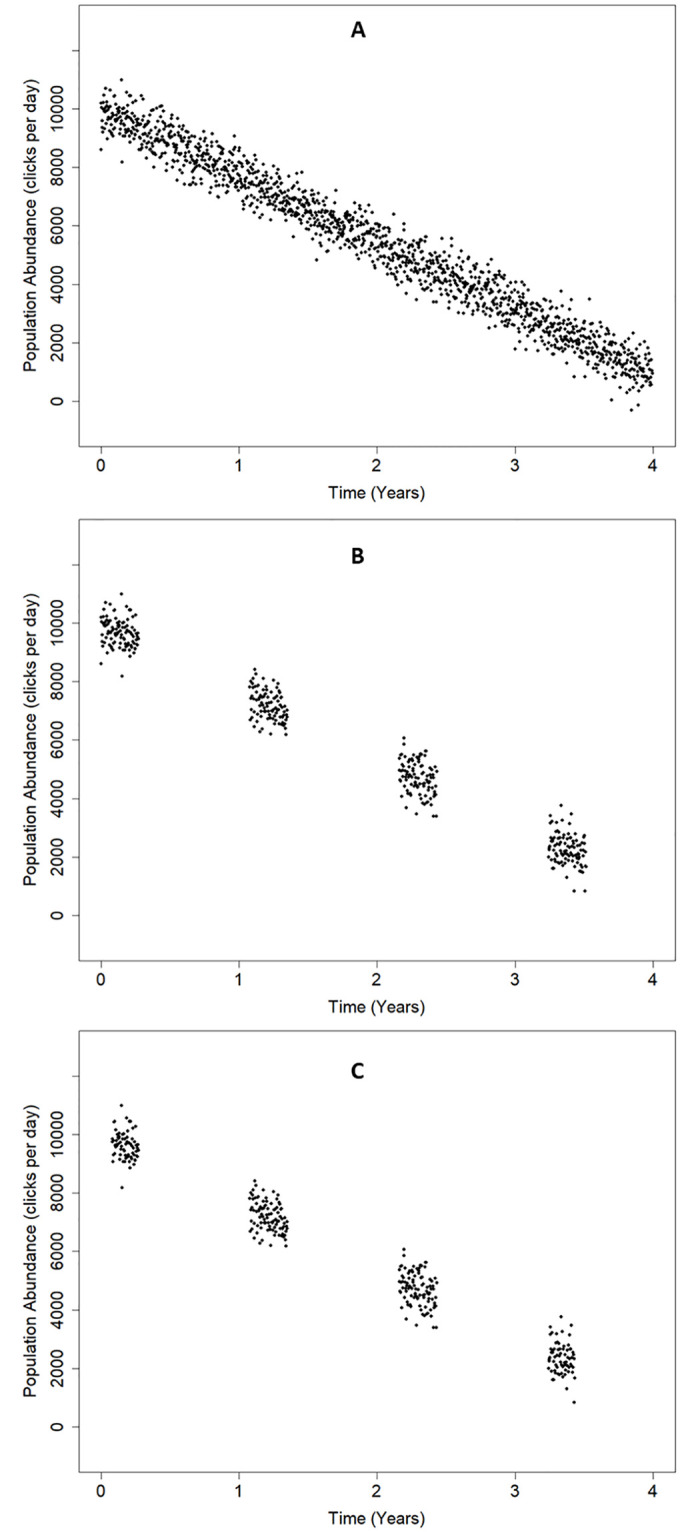
Scenario 2: Uniform downward population trend. (A) *complete data*, (B) *incomplete data* and (C) *paired data*.

The population starts at the same level as in Scenario 1 but has a continuous linear decline that is not seasonal and continues across years, as shown in Fig 2.

Three data sets were extracted from each scenario: *complete data*, *incomplete data* and *paired data*. *These* are shown in Figs 1 and 2 and are:

*Complete data*. All days are ‘fully logged’ so the trend analysis is based on 4 x 365 days as we omit the extra day from leap years from the analysis.

*Incomplete data*. Data were drawn from a 100-day window that ‘shifted’ forwards by 30 days each year, giving only 27% (100% x 400/1460) of the *complete data* and reducing the temporal overlap between successive years to 70 (100–30) paired days, with only 10 [100- (3x30)] specific days logged in all 4 years.

*Paired data*. These are the data from the incomplete data set that are used by PYRA and consists of the logged days in any 365-day period that were also logged (i.e. they have matching day numbers within the 365-day period) in the succeeding 365-day period. This set has 340 days (23% of the total).

### Real data: Harbour porpoises and orcas

**Fig 3:**
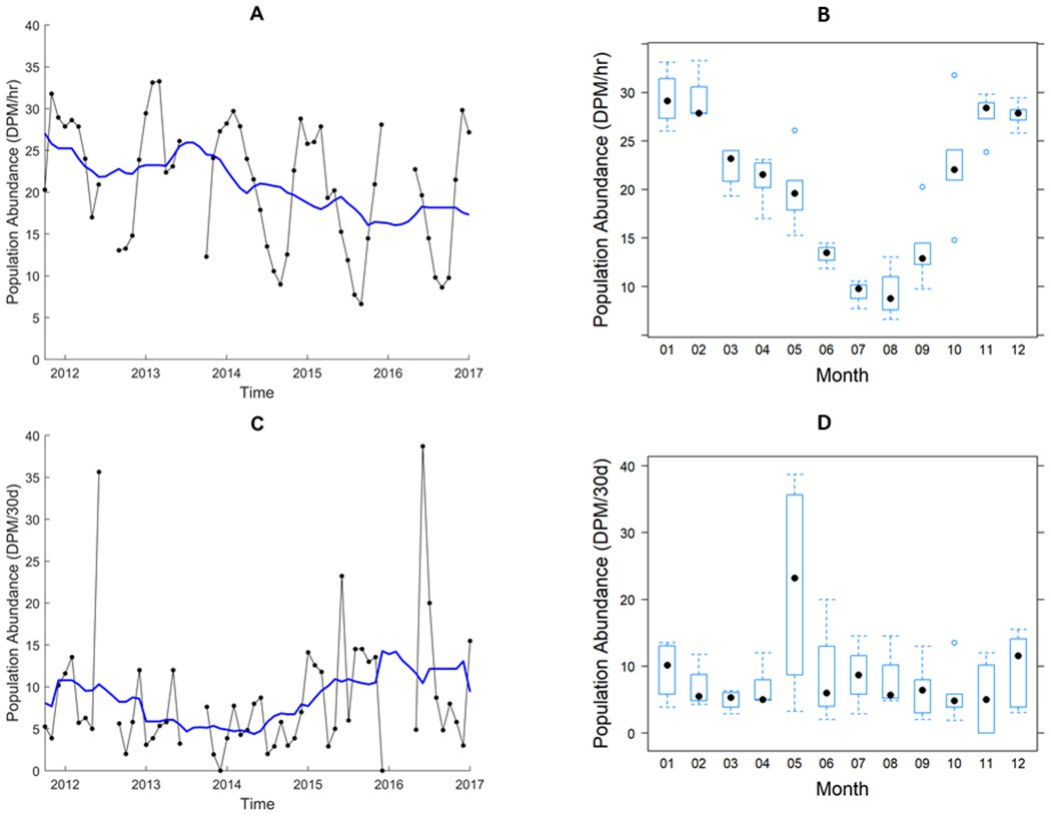
Data recorded from Burrows Pass, USA, 15^th^ September 2011 to 15^th^ December 2016 and aggregated into 30-day periods. (A) with a 1-year moving average superimposed (solid blue line) For harbour porpoises as the mean number of detection positive minutes per hour (DPM/hr) and (C) for orcas as the mean number of detection positive minutes per 30 days (DPM/30d), with corresponding boxplots (B,D) to show seasonal patterns. Over the time span of the data set there were 35,915 detection hours in which a total of 735,776 and 431 detection positive minutes were recorded for harbour porpoises and orcas respectively.

As static acoustic monitoring technology has been used mainly for the study of population impacts and distribution, examples of trend studies are very limited. Here we illustrate the application of PYRA to real data obtained from a single C-POD acoustic monitor (manufactured by Chelonia Ltd., Mousehole, Cornwall, UK, http://www.chelonia.co.uk, for example, see [3]) which was deployed over 64 months (5.33 years) between September 2011 to December 2016 in Burrows Pass in the Salish Sea (a marginal sea of the Pacific ocean) in Washington State, USA at 48°29’18.13"N 122°41’13.56"W. Data gaps amounted to 9 of the 64 months. The species known at this site are the harbour porpoise *Phocoena phocoena* and orca *Orcinus orca*. The porpoise population is declining in many locations worldwide [4,18,19]. It is known to be changing dynamically in the Salish Sea [20–23], with the initial population decline likely due to gill net fishing and to pollution. From around the year 2000, the porpoise population appeared to rebound but since 2010, large numbers of transient orcas have returned regularly to predate on harbour seals and porpoise. Within the same time frame, grey and humpback whales, two species mostly absent before 2000, also returned in large numbers to consume large volumes of forage fish making up the harbour porpoise diet. From extensive visual observations, an emerging conjecture is that the increase in orca (as predators) combined with a loss of forage fish (as prey) may have had a negative impact on the Salish Sea harbour porpoise population. Therefore, the experimental objective of the static acoustic monitoring was to determine whether the harbour porpoise population trend in the Salish Sea is either increasing or decreasing. The site selected for the C-POD location was assessed to be a stronghold for the porpoise population with the benefit of also allowing land-based observers to record and verify porpoise presence. Fig 3 shows the monthly values and a moving 1-year average.

### Paired Year Ratio Assessment (PYRA)—Informal description

We have developed PYRA for the type of time series data obtained from static acoustic monitors at one or more sites in order to provide a measure of the proportional change occurring in the detection rate of a species between successive years.

The detection metric can be chosen according to the judgement of the user; the number of detection positive minutes per day (DPM/day) has been used in many studies, as has the total number of clicks logged. The former entails a risk of encountering saturation of the metric, and the latter entails some risk of conflating cetacean behaviour with presence of cetaceans as clicks are produced more rapidly during some social and foraging activities. Other metrics, such a detection positive seconds could also be used.

The process applied is:

1. Comparisons are made only between one annual period (termed the first year, Y1) and the following annual period (termed the second year, Y2), using only the data from those whole days at each site, specified by their position within the annual cycle, which were fully logged in both the first and second years—we refer to these as ‘paired data’. At some sites many, or all, of the paired days may have no detections recorded.
2. The ratio of the sum of the detections at a site in the second year divided by the first is treated as the year-on-year ratio i.e., the annual proportional change at the end of the first year for that site.
3. Where multiple sites have paired data in a 730-day (2 year) period, data from the sites are combined by summing the detections for year 1 across all sites. The same is done for year 2 and a global ratio is can then be obtained.
4. If more than two years of data are available at any site, this two-year data window is moved forwards one day at a time, and process is repeated.
5. A measure of uncertainty is obtained by random resampling of the paired data, with replacement, to give a distribution of ratio values. If the data consists of many sites with substantial detection rates, the range of uncertainty around the ratio will be lower than where many sites have low detection rates.

There are strengths and weaknesses to this approach that we discuss later.

### Paired Year Ratio Assessment (PYRA)—Formal description

PYRA provides a statistical estimator $\hat{P}$ to describe the year-on-year proportional change in counts of detections between successive years Y_1_ and Y_2_ derived over the time span T of the data set. Here we use ‘year’ to refer to the duration of the periodic cycle of relevance to the data under consideration because typically this will be of annual duration for cetaceans. However, the periodic cycle duration could, in general, be of any duration (for example, a day) deemed appropriate for the data. As a ratio, $\hat{P}$ is an increasingly unstable statistical estimator as the count in year Y_1_ or year Y_2_ approaches zero. It will be undefined at any time points when observations were recorded as zero by the acoustic monitor in the denominator year Y_1_. This can be adjusted by inserting a small positive value, as is typically done when transforming data *x* to a logarithmic (1+*x*) scale, or with other unstable ratio statistical estimators such as the standardised incidence rate employed in spatial epidemiology [24]. However, in practice, smoothing of the data will generally be required such as through aggregating data by a moving average window.

We denote the data recorded at time *t*_*k*_ at the monitoring site as *y*_*k*_ so that the set of all data points {(*t*_*k*_, *y*_*k*_)} is a time series. The collected data in the baseline year Y1 is denoted by *Y*_1_{(*t*_*k*_, *y*_*k*_)} and the paired data in year Y1, $Y_{1}\left\{\left(t_{k}^{*},y_{k}^{*}\right)\right\}$ is defined as the subset of *Y*_1_{(*t*_*k*_, *y*_*k*_)}, such that for every $y_{k}^{*}$ at time $t_{k}^{*}$ in Y1 there exists a corresponding $y_{k+C}^{*}$ at time $t_{k+C}^{*}$ in the following year Y2, denoted by $Y_{2}\left\{\left(t_{k+C}^{*},y_{k+C}^{*}\right)\right\}$ where C is a constant defining the duration of an annual cycle in the selected time units. For example, with daily incremental data, C = 365 days, while with monthly incremental data C = 12 months. Therefore, the set of data points $Y_{1}\left\{\left(t_{k}^{*},y_{k}^{*}\right)\right\}$ in the baseline year Y1 are paired with corresponding data points $Y_{2}\left\{\left(t_{k+C}^{*},y_{k+C}^{*}\right)\right\}$ in the following year Y2. If all the times within the year at which data were recorded in Y1 and Y2 are identical then clearly the paired data $\left\{Y_{1}\left\{\left(t_{k}^{*},y_{k}^{*}\right)\right\}\;U{\;Y}_{2}\left\{\left(t_{k+C}^{*},y_{k+C}^{*}\right)\right\}\;\right\}$ are identical to the original data set {(*t*_*k*_, *y*_*k*_)}. However, because in practice data gaps in successive years will often not correspond the paired data set will often be a subset of the original data.

We define PYRA for paired data sets $Y_{1}\left\{\left(t_{k}^{*},y_{k}^{*}\right)\right\}$ and $Y_{2}\left\{\left(t_{k+C}^{*},y_{k+C}^{*}\right)\right\}$ at time $t_{k}^{*}$ by the estimator ${\hat{P}}_{I}\left(t_{k}^{*}\right)$

$${\hat{P}}_{I}\left(t_{k}^{*}\right)=\;m_{I}\left(y_{k+C}^{*}\right)/m_{I}\left(y_{k}^{*}\right),$$ (1)

where *m*_*I*_(*y*) is a smoothing function, such as a forward moving average with window span of width I, operating over a sequence of successive year pairs. The purpose of the smoothing function *m*_*I*_(*y*) is to mitigate any bias or inflation occurring in trend estimation from either the presence of noise, or if the denominator term is zero, or when gaps in the collected data would otherwise result in ${\hat{P}}_{I}\left(t_{k}^{*}\right)$ being undefined. The average value of ${\hat{P}}_{I}\left(t_{k}^{*}\right)$ over the time-span T of the data set is a summary trend metric defined by

$$\tilde{P_{I}}\;=\left(\frac{1}{K-I+1}\right)\overset{k=K-I+1}{\underset{k=1}{\sum }}\left(\;m_{I}\left(y_{k+C}^{*}\right)/m_{I}\left(y_{k}^{*}\right)\right)$$ (2)

where $\tilde{\left[X\right]}$ denotes the sample mean of *X* and *K* is the total number of paired data points within the time span T of the data set.

Typically for cetacean data collected over a period of years, the natural choice for the span width I in Eq (1) is the duration of the annual cycle C, giving rise to the metric ${\hat{P}}_{C}\left(t_{k}^{*}\right)$ which, by a shift of C/2 time units to the right becomes ${\hat{P}}_{C}\left(t_{k+C/2}^{*}\;\right),$ in order to remove the time lag of C/2 units resulting from the PYRA forward sliding window. The plot of ${\hat{P}}_{C}\left(t_{k+C/2}^{*}\right)$ against time over the time span of the data set shows how the trend fluctuates and is referred to as a PYRA population trend plot.

In Eq (1), if the span width I is set at zero, we obtain the PYRA point estimator which for conciseness of notation we denote by

$$\hat{P}\left(t_{k}^{*}\right)=y_{k+C}^{*}/y_{k}^{*}\;.$$ (3)

In Eq (2), If the span width I is set at the time span T of the data set, then we obtain the summary PYRA trend statistic for the time series

$$\tilde{P_{I}}={\tilde{P}}_{T}=\;{\hat{P}}_{T}\left(t_{k}^{*}\right).$$ (4)

The trend statistic ${\hat{P}}_{T}\left(t_{k}^{*}\right)$, which we abbreviate to ${\hat{P}}_{T}$, gives an average measure of the trend over the time span T of the data set. Specifically, $100\%\;X\;(1-{\hat{P}}_{T})/T$ quantifies the trend as an average year-on-year percentage change based on the paired data within the whole time-span T of the data set. If the proportional decreases are balanced by the proportional increases, then ${\hat{P}}_{T}=1$ which provides a useful ‘no trend’ baseline.

An extension of the approach to determine a regional PYRA from data collected from multiple acoustic sites is provided in the S1 Appendix.

### Incorporation of PYRA uncertainty

The uncertainty associated with ${\hat{P}}_{C}\left(t_{k}^{*}\right)$ and ${\hat{P}}_{T}$ is estimated through a moving block bootstrap approach [12,25,26], defined here by a short time window spanning *w* time units and centred at the midpoint which slides along the paired data sets $Y_{1}\left\{\left(t_{k}^{*},y_{k}^{*}\right)\right\}$ and $Y_{2}\left\{\left(t_{k+C}^{*},y_{k+C}^{*}\right)\right\}$. The value of *w* must be chosen so that there will be little correlation between the first and last observations in the window. The approach works by randomly resampling the consecutive sets of *w* observations defined by the sliding window, several times (typically 1000), then importing each resample into the smoother function *m*(y) for the PYRA calculation. This process generates a distribution of PYRA values which are used to derive a 100(1-α)% percentile confidence interval at a desired significance level of α% [e.g. 12,26,27].

### Randomisation trend tests

Data collected by acoustic monitors are a set of ordered observations in which each observation has an associated observation time. The data are thus a time series, with the inherent property that observations are not interchangeable unless the observation values are completely time independent of each other. One way of testing for a trend in a time series is through a randomisation trend test which assesses whether the observed data are statistically significantly different from the null hypothesis of no trend [12,25,28]. The alternative hypothesis is that there is a trend present. In common with PYRA, randomisation trend tests make no assumption about an underlying model and are nonparametric. They proceed by asserting that the observed time series is a random permutation drawn from the random distribution of all possible permutations of the time series. The presence of any gaps or missing data therefore does not affect their utility. The randomisation test outcome is based on comparing a relevant test statistic evaluated for the original time series with its respective randomisation distribution [12]. We applied four well known nonparametric trend Randomisation Tests (RT) to the time series data sets {(y_t_, t)} defined as follows:

RT1. The ‘linear trend test’ with the regression coefficient *m* (in the regression model y = *m*x + *c*) taken as the test statistic. A significantly negative or positive value for *m* respectively indicates a downward or upward trend.
RT2. The ‘runs above and below the median’ test, where the test statistic is the number of runs above and below the median. A ‘run’ is defined as a successive sequence of values {y_i_} that are either above or below the median. A significantly low number of runs indicates a trend (because longer runs lead to a lower number of runs overall).
RT3. The ‘signs test’ where the statistic is the number of positive differences (y_i+1_ –y_i_) calculated between all the successive data points y_i_ and y_i+1_. A significantly low number of positive differences indicates a downward trend and a significantly high number indicates an upward trend.
RT4. The ‘runs up and down test’ where the test statistic is the total number of runs of either positive or negative differences as defined previously in RT3. A significantly low number of runs indicates a trend.

For a trend test to be effective, it must properly account for intra-annual or inter-annual fluctuations which typically occur in cetacean populations. In each randomisation test, the randomisation distributions were generated by drawing 5000 random samples from the data set.

### General additive modelling

General additive models (GAMs) have been widely applied to a variety of observational data sets with the purpose of assessing trends in ecological populations [11,17,19]. A GAM is a Generalised Linear Model (GLM) in which the linear predictor G(X_i_) depends linearly on smoother functions s_i_ of predictor variables X_i_, but whose exact parametric form is unknown [17]. As with a GLM, a third and final component to be specified is a link function L defined by L[G(X_i_)] = E[Y_i_] which maps the linear predictor to the expected value E[Y_i_] of the observational data Y_i_. A GAM is a sophisticated nonparametric regression model which utilises a set of nonlinear basis functions to determine the smoothers and then arrive at an optimal fitted curve to the data as a sum of the smoothers, through employing penalty terms for overfitting. Here we employ a GAM as a benchmark for comparison of PYRA performance in trend estimation with each of the above data sets. The GAM has up to two smoother functions to account for inter-annual or intra-annual fluctuations in the data and is specified by

$$\text{G}\left({\text{X}}_{\text{i}1},\;{\text{X}}_{\text{i}2}\right)=\alpha +{\text{s}}_{1}\left({\text{X}}_{\text{i}1}\right)+{\text{s}}_{2}\left({\text{X}}_{\text{i}2}\right)+\varepsilon_{\text{i}}$$ (5)

where, α is an intercept, s_1_ and s_2_ are smoother functions of the respective explanatory variables X_i1_ for *year*, Xi_2_ for *day of year* and error *ε*_i_ assumed to be gaussian identically independently distributed with ε_*i*_ *~* N (0, σ^2^) with σ^2^ constant variance, referred to as homogeneity. The link function L used here is the identity link so that L[G(X_i1_, X_i2_)] = G(X_i1_, X_i2_) = E[Y_i_] where y_i_ are the click data. The model was fitted with s_1_ and s_2_ as penalized cubic regression splines with basis dimension 5 using the *mgcv R* package [17].

## Results

PYRA was applied to each data set and the average trend statistic ${\hat{P}}_{T}$ together with PYRA Population Trend plots for ${\hat{P}}_{C}\left(t_{k}^{*}\right)$ and 95% percentile confidence limits [L = lower, U = upper] were determined. The estimated trend in each case was compared with results obtained with the four nonparametric randomisation trend tests and also with smoothers determined by the GAM defined in Eq (5).

### PYRA and randomisation trend tests

#### Synthetic data

**Fig 4:**
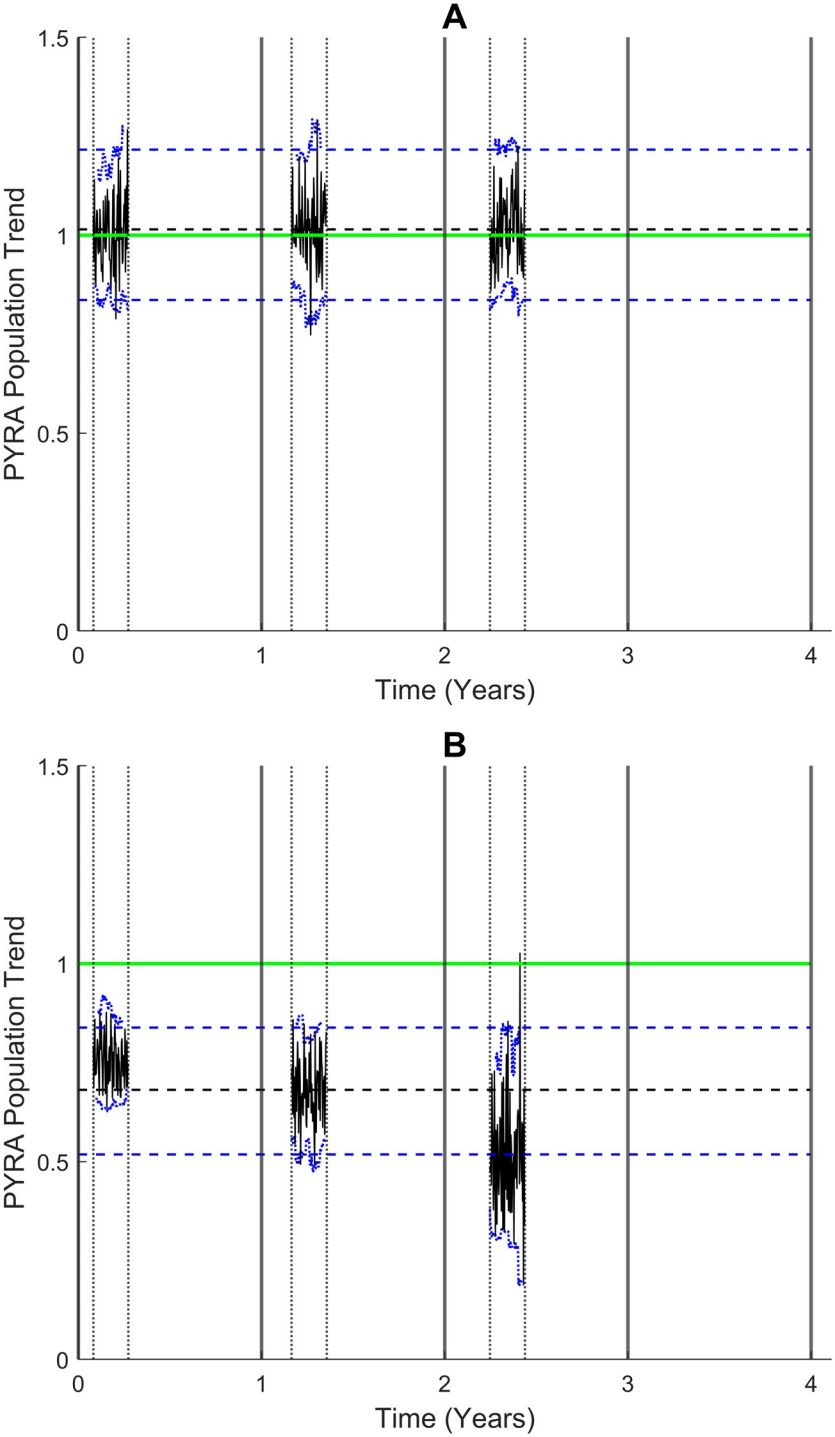
The PYRA statistical estimator $\hat{P}(t^{*})$ (solid black) for the *paired data sets* plotted against time with 95% percentile lower (L) and upper (U) confidence limits (dotted blue) computed with a sliding bootstrap window of width *w* = 21 days (3 weeks) for (A) Scenario 1 and (B) Scenario 2. The respective trend mean averages $\tilde{\left[\hat{P}\left(t^{*}\right)\right]=}{\hat{P}}_{T}$ over the total time span T of the data set are superimposed (dashed black) together with 95% percentile confidence limits (dashed blue). The baseline ‘no trend’ PYRA value of 1 is the horizontal green line. In (A) the trend statistic $\hat{P_{T}}=1.006$ (L = 0.947, U = 1.013) indicates the trend is flat; in (B) $\hat{P_{T}}=0.676$ (L = 0.647, U = 0.699) indicates a downward trend, estimated at 33% [(1–0.676) x 100%] over the time span T of the data set.

Fig 4 shows PYRA population trend plots for (A) Scenario 1 and (B) Scenario 2 evaluated using their respective paired data sets. In both cases these confirm that PYRA correctly identified the underlying patterns enforced by design into each scenario over the whole-time span of the data.

Table 1 compares the PYRA results together with those of the 4 randomisation trend tests applied to each of the categorised data sets. As a hypothesis test, a randomisation test only provides the displayed p-values thereby enabling statistical significance to be evaluated. However, PYRA provides a quantifiable trend estimate together with an associated uncertainty in the form of a percentile confidence interval [L, U], as shown in the rightmost column.

**Table 1:** Comparison of PYRA trend statistic $\hat{P_{T}}$ with four randomisation trend tests applied to the synthetic data.

	Randomisation trend test								PYRA
	RT1		RT2		RT3		RT4		$\hat{P_{T}}$
**Scenario 1 data**	Stat *m*	p value	Stat	p value	Stat	p value	Stat	p value	
Complete	-1.489	< 0.0001	96	< 0.0001	948	0.0554	697	0.0002	N/A
Incomplete	-1.906	< 0.0001	70	< 0.0001	258	0.1824	192	0.1136	N/A
Paired	-1.389	< 0.0001	70	< 0.0001	218	0.1564	164	0.1766	1.006 (0.947–1.013)
**Scenario 2 data**									
Complete	-6.230	< 0.0001	90	< 0.0001	958	0.1848	726	0.3850	N/A
Incomplete	-6.271	< 0.0001	2	< 0.0001	256	0.1198	199	0.5010	N/A
Paired	-6.311	< 0.0001	2	< 0.0001	217	0.1320	171	0.4304	0.676 (0.647–0.699)

The sample statistic *Stat* was determined by the randomisation test and is shown with p-values for each data set. RT1: Linear regression with the regression slope coefficient *m* as the sample statistic; RT2: Number of runs above or below the median value of the data; RT3: Number of runs of positive or negative differences; RT4: Count of positive or negative differences between consecutive data points. $\hat{P_{T}}$ with 95% percentile confidence limits (lower, upper) and statistical significance at the 0.01% significant level. 5000 randomisations were used to generate the randomisation distributions and percentile confidence intervals throughout.

#### Scenario 1

PYRA accurately indicated that the population was stable with no detectable trend over the whole- time span of the data set, as indicated by the PYRA trend statistic ${\hat{P}}_{T}=1.005$ (L = 0.947, U = 0.1.013) proximity to the baseline value of 1. However, randomisation tests RT1 and RT2 incorrectly indicated a highly statistically significant downward trend (p < 0.0001) using each of the categorised data sets. The test RT3 correctly indicated no statistically significant trend using each categorized data set, as did test RT4 with the incomplete and paired data sets. Test RT4 incorrectly indicated a statistically significant trend (p < 0.001) with the complete data set.

#### Scenario 2

PYRA accurately indicated a strong downward trend with ${\hat{P}}_{T}=0.676$ [L = 0.647, U = 0.699] and thus that the year-on-year abundance decreased by an estimated 32% [(1–0.676) x 100%] over the whole- time span of the data set. Although randomisation tests RT1 and RT2 correctly indicated a highly statistically significant downward trend (p <0.0001) using each of the categorised data sets, tests RT3 and RT4 failed to determine any statistically significant trend in all cases.

In summary, the outcome of the randomisation tests demonstrates that the statistical significance attached to an estimated trend may be strongly influenced by localised short-term fluctuations. This meant that trend assessment was correct only 50% of the time, namely in 2 out of the 4 tests in each scenario. False positives (Type 1 Error) occurred when no trend was present in Scenario 1 (using tests RT1 and RT2) whilst false negatives (Type 2 Error) were produced when a trend was present in Scenario 2 (using tests RT3 and RT4). In direct contrast, the PYRA approach yields consistent trend assessments in both scenarios. Furthermore, PYRA offers an ecologically interpretable measure of effect size associated with any trend assessment.

The four randomisation trend tests were repeated for each scenario with lower or higher levels of random variation in the data and results were again compared with trend assessment by the PYRA approach. The results similarly confirmed the robustness of PYRA (Supporting Information S2 and S3 Figs, S1 Table).

#### Harbour porpoises and orcas data

**Fig 5:**
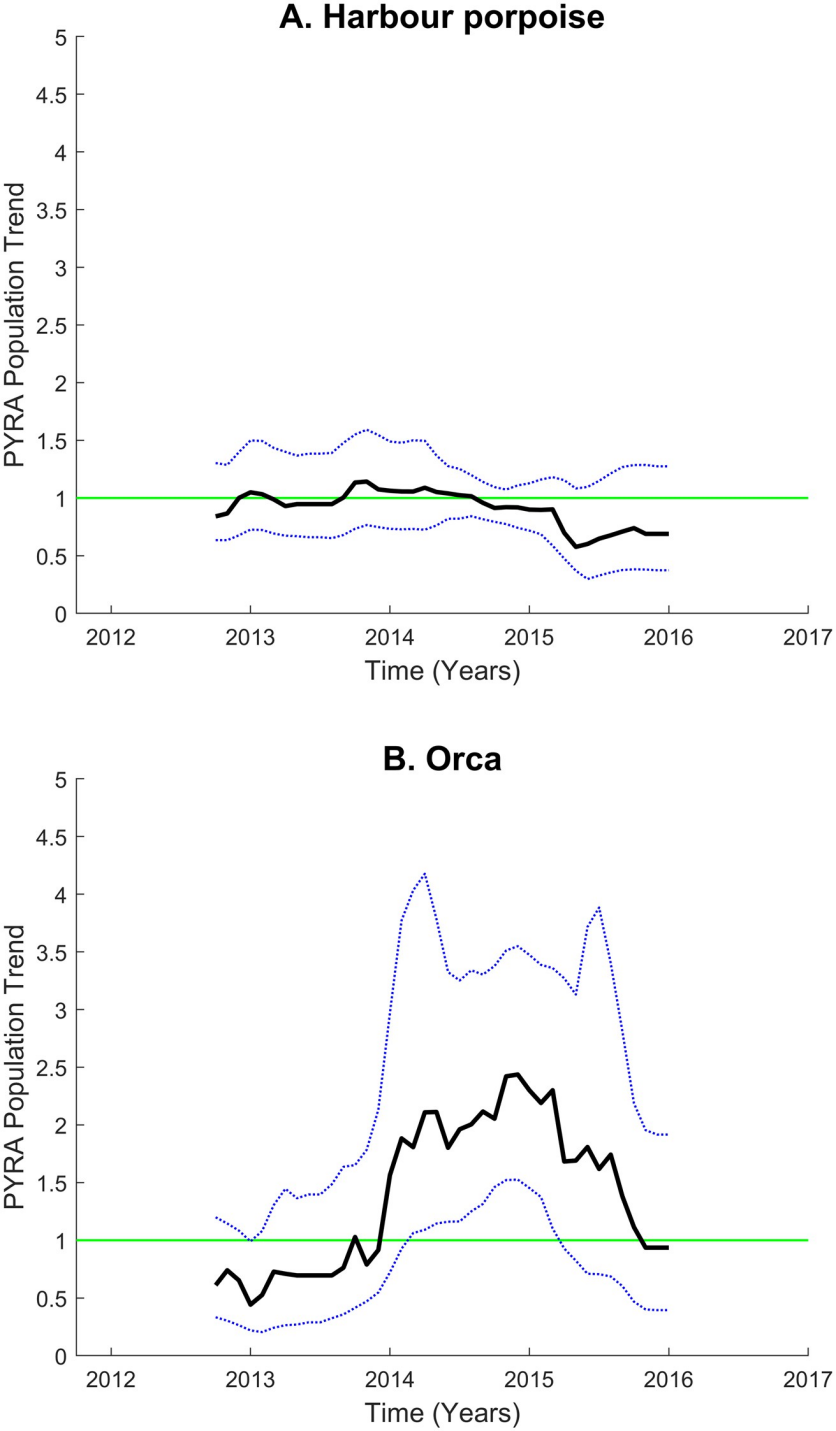
PYRA population trend plots showing $\hat{P_{C}}(t^{*})$ against time (solid black) with 95% percentile lower (L) and upper (U) confidence limits (dotted blue) computed with a sliding bootstrap window of width *w* = 3 months (90 days) over the time span T of the Salish Sea data set for harbour porpoises (A) and orcas (B). The baseline ‘no trend’ PYRA value of 1 is the horizontal green line. (A) harbour porpoises show a recent downward tendency, the trend statistic $\hat{P_{T}}=0.928$ (L = 0.886, U = 0.990) indicates an overall downward trend. (B) orcas show an upward followed by recent downward fluctuation, the trend statistic $\hat{P_{T}}=1.189$ (L = 0.962, U = 1.433) indicates an overall upward trend but with high uncertainty.

(Fig 5A and 5B) shows the respective PYRA population trend plots determined for harbour porpoises and orcas from their *paired data* sets. The relatively flat PYRA plot in Fig 5A for harbour porpoises indicates that the year-on-year fluctuations visible in the time series plots of the data (Fig 3A) were relatively stable. However, the PYRA trend statistic ${\hat{P}}_{T}=0.928$ (0.866–0.990) indicates a downward trend for this species over the time span of the data set. For orcas, the PYRA population trend plot in Fig 5B shows an upward trend which peaks in the central point of the observation period. The PYRA trend statistic ${\hat{P}}_{T}=1.189$ (0.962–1.433) indicates that over the time span of the data the general overall trend is upward but with wide confidence limits implying high uncertainty.

The results in Table 2 show that randomisation tests performed more consistently with real data than with the synthetic data sets, indicating a downward trend for harbour porpoises that was statistically significant (p < 0.01) in test RT1, and highly statistically significant (p < 0.0001) in tests RT2 and RT3. For orcas, although an upward trend was suggested, this tendency was not statistically significant in any of the four tests. These results concur overall with the above PYRA population trend estimates for both species shown in the rightmost column. The PYRA trend statistic ${\hat{P}}_{T}$ for harbour porpoises indicates an average decline of 7% [(1–0.928) x 100] with the narrow confidence limits implying strong certainty. In contrast, for orcas, the PYRA trend statistic indicates an average increase of 19% but with high uncertainty implied by the wide span of the associated confidence limits.

**Table 2:** Comparison of PYRA trend statistic $\hat{P_{T}}$ with the four randomisation trend tests applied to the Salish Sea data set.

	Randomisation trend test								PYRA
	RT1		RT2		RT3		RT4		$\hat{P_{T}}$
**Salish Sea data**	Stat *m*	p value	Stat	p value	Stat	p value	Stat	p value	
Porpoises (*n = 55*)	-0.137	0.004	14	< 0.0001	22	< 0.0001	27	0.584	0.928 (0.886–0.990)
orcas (*n = 55*)	0.063	0.127	26	0.297	32	0.106	30	0.096	1.189 (0.962–1.433)

RT1: Linear regression with the regression slope coefficient *m* as the sample statistic; RT2: Number of runs above or below the median value of the data; RT3: Number of runs of positive or negative differences; RT4: Count of positive or negative differences between consecutive data points. $\hat{P_{T}}$ with 95% percentile confidence limits (lower, upper) and statistical significance at the 0.01% significant level. 5000 randomisations were used to generate the randomisation distributions and percentile confidence intervals throughout.

### General additive modelling

To compare the performance of the PYRA approach directly with GAM, we fitted the GAM model defined in Eq (5) to the *paired data*, that is, the identical data required by PYRA, obtained for each of the data sets. Arguably, a comparison of GAM should also be made using the *incomplete data* since a larger data set may be more informative when modelled via GAM. The GAM model was therefore also fitted to the respective *incomplete data* sets obtained in each case and results were similar throughout (Supporting Information S2 Appendix and S3 Figures (3–4) in S1 Fig).

### Synthetic data

#### Scenario 1 and Scenario 2

**Fig 6:**
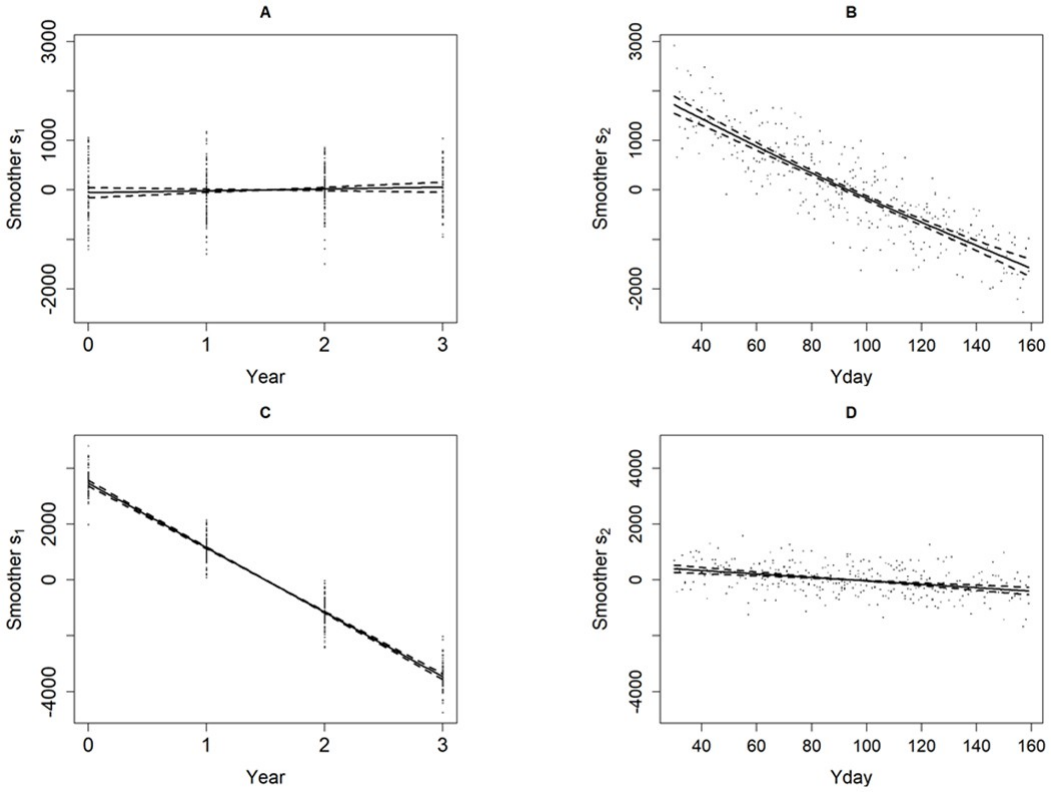
Smoothers (solid line); *s*_1_ for year and *s*_1_ for day within the year (Yday), with 95% confidence bands (dashed) and partial residuals (dots) obtained by fitting the GAM model to the *paired data* of (A-B) Scenario 1 and bottom row (C-D) Scenario 2. For Scenario 1, the plots respectively indicate in (A) no long-term trend (p = 0.282), in (B) a seasonal decline which is highly statistically significant (p = 2e-16). Conversely, for Scenario 2, in (C) the long-term decline trend is highly statistically significant (p = 2e-16), in (D) the seasonal linear downward trend is highly statistically significant (p = 4.05e-09). The fitted GAM model diagnostics were reasonable for both Scenarios (see Supporting Information S3 Figures (1–2) in S1 Fig).

Fig 6 compares plots of the smoothers s_1_ and s_2_ obtained from fitting the GAM model to the *paired data* of each scenario. The corresponding GAM diagnostic plots are shown in the Supporting Information S3 Figures (1–2) S1 Fig.

For Scenario 1 the smoother curve s_1_ in Fig 6A indicated no statistically significant trend over the time span of the data set (p = 0.282), while the smoother curve s_2_ in Fig 6B indicated the presence of a highly significant periodic component within the year (p < 0.0001). For Scenario 2 the smoother s_1_ in Fig 6C indicates a highly statistically significant downward trend over the time span of the data set (p <0.0001), while the smoother s_2_ in Fig 6D also indicates a highly statistically significant annual downward trend (p< 0.0001). Similar results were obtained from fitting the GAM to the *incomplete data* set and these are provided in the Supporting Information S2 Appendix. The PYRA trend assessments for both Scenarios were therefore found to be consistent with those achieved by fitting the appropriate GAM model.

GAM analyses were similarly repeated for each scenario with lower or higher levels of random variation in the data and results were again compared with trend assessment by the PYRA approach. The results similarly confirmed the robustness of PYRA (Supporting Information S2 and S3 Figs, S1 Table).

#### Harbour porpoises and orca data

**Fig 7:**
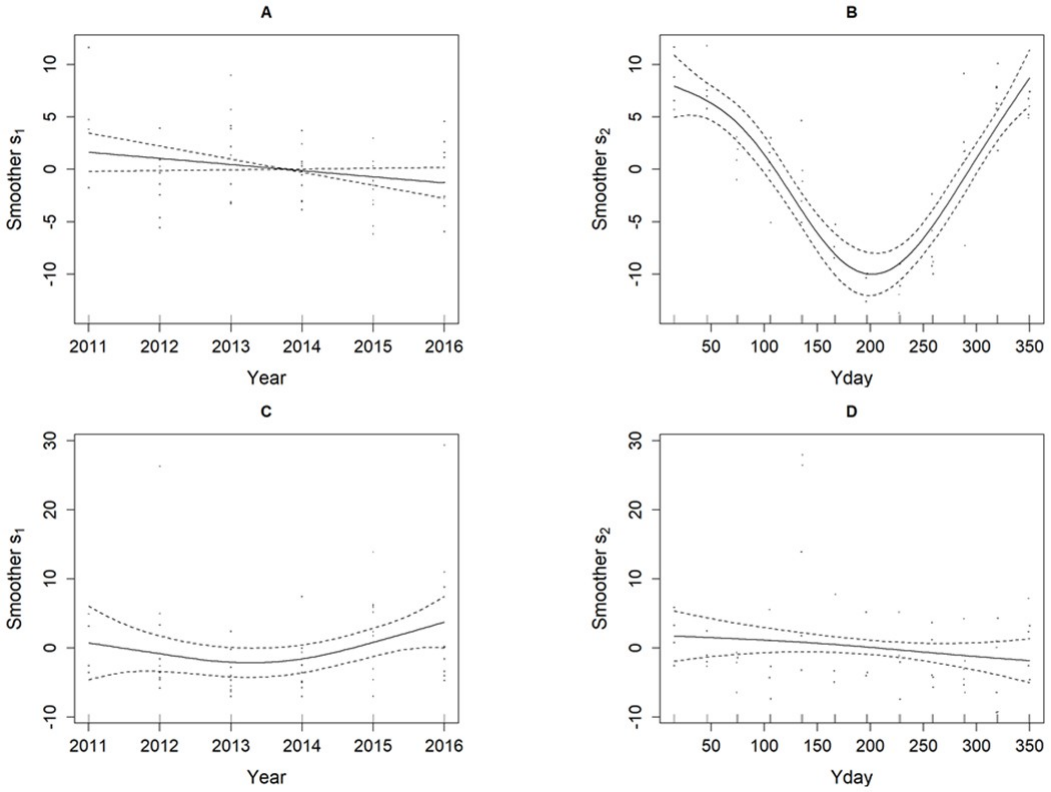
GAM smoothers (solid line) *s*_1_ for year and *s*_2_ for day within the year (Yday), with 95% confidence bands (dashed) and partial residuals (dots) obtained by fitting the GAM model to the Salish Sea data for (A-B) harbour porpoises and (C-D) orcas. For harbour porpoises, (A) ***s***_**1**_ shows a slight downward long-term trend which is not statistically significant (p = 0.0826); (B) ***s***_**2**_ shows a deep seasonal trough which is highly significant (p < 2e-16). For orcas, (C) ***s***_**1**_ shows an upward long-term trend (p = 0.145); (D) ***s***_**2**_ shows a seasonal downward decline (p = 0.310), neither of which are statistically significant. The fitted GAM model diagnostics were reasonable for harbour porpoises but weak for orcas (Supporting Information S3 Figures (5–6) in S1 Fig).

Fig 7 shows the plots of smoothers s_1_ for Year and s_2_ day of year (Yday) obtained for the GAM from model fitting to (AB) harbour porpoises and (CD) orcas.

For harbour porpoises, the smoother s_1_ for year shown in Fig 7A indicates a slight downward trend which is not statistically significant over the observation period (p = 0.0826), while the smoother curve s_2_ of Fig 7B for within the year fluctuations confirmed the presence of a highly significant seasonal component (p<0.0001) as can be seen by visual inspection of the data in Fig 3B. Model diagnostics indicated the model was a reasonable fit (Supporting Information S3 Figure 5 in S1 Fig). For orcas, the smoother s_1_ for year shown in Fig 7C displayed a trough in the central part of the trend but this was not significant (p = 0.145). Similarly, the smoother curve s_2_ shown in Fig 7D suggested a periodic component within the year but this was also not significant (p = 0.310). However, the model diagnostics indicated the model provided a poor fit (Supporting Information S3 Figure 6 S1 Fig).

In summary, the estimated trends over the timespan of the data set for each species inferred from the GAM smoother s_1_ in each Scenario are broadly in line with those obtained with PYRA, indicating a significant downward trend for harbour porpoises but with no clear or significant trend inferable for orcas.

## Discussion

In recent years static acoustic monitoring has succeeded in cetacean monitoring tasks that were beyond the reach of other methods. In the SAMBAH project [29], 300 site-years of data were collected from 200 sites across the Baltic Sea and have transformed knowledge of the elusive Baltic Sea harbour porpoise. In the Vaquita monitoring project in Mexico, static acoustic monitoring has been the basis for tracking the tragic decline of the last tiny population of *Phocoena sinus* [3]. Both these projects used sophisticated analysis that represent ideal approaches, and both indicate that this form of monitoring has a valuable future role.

PYRA is designed as a descriptive exploratory tool for estimating trends from such data where it extends over multiple years. It provides a quantitative description of how a cetacean population trend fluctuates year-on-year, over the monitored timespan. These estimates are readily interpretable and the uncertainty around them can be quantified. The trend estimation is achieved by making nonparametric year-on-year evaluations with only the recorded data in hand, without a need to quantify diel or intra-annual or seasonal variations as separate components.

Our approach contrasts with GAM where the user must *a priori* specify a seasonal component if one is to be incorporated into the trend estimation process, as with the s_2_ term included in the GAM model of Eq (5). As a more sophisticated approach, GAM permits refined analyses to be carried out, but only if the researcher (and ultimately reader) has the necessary knowledge and model interpretive skills. Contrastingly, PYRA is simple to understand and gives results that are readily interpreted and accessible to statistically less expert researchers and could act as a precursor to a more sophisticated analysis if required.

The estimation of the uncertainties of the PYRA outputs can easily be related to their biological significance. In many other simple testing approaches, for example, with a randomisation trend test, biological and statistical significance are hard to disentangle. In that case, failure to reject the null hypothesis does not mean that there is no trend, just that there was a lack of statistical power to detect it. Conversely, in a large study one might detect statistically significant but ecologically weak effect sizes and conflate one with the other.

The effectiveness of GAM smoothing for the purpose of stabilisation of ${\hat{P}}_{I}\left(t_{k}^{*}\right)$ will necessarily be influenced by the amount and pattern of missing data. In particular, smoothing may introduce a bias into an estimated time series trend when the proportion of missing data is high. An alternative option in this situation is to apply an imputation method to assign estimated values to the missing data [30]. The data recorded by multiple site monitors equate to multivariate time series so that multiple imputation methods such as MICE may be applied to infer any missing values [31]. In the case of single acoustic monitor site data, univariate imputation methods ranging from linear interpolation to Kalman smoothing may be used. However, there is no general consensus in the literature on what the maximum proportion of ‘missingness’ should be for such approaches to be demonstrably efficient [32,33]. Consequently, application of such approaches requires considerable skill and experience.

The key practical constraints that field biologists need to recognise come from some factors that affect all forms of point monitoring and some factors that are specific to acoustic monitoring.

Factors affecting any type of point monitoring:

- Few detections: this situation gives high sampling error and wide confidence intervals.
- Large data gaps, irregularly distributed: these can distort results from most methods.
- Changes in logging stations: any change that is liable to affect detection rates would require the site data to end and restart as a new site.
- Representativeness of sites: if monitoring is intended to reflect changes within some area, rather than at one location, then sites in a range of habitat types within that area are needed, if they exist. If differing trends are seen across different habitat types, then some assessment of redistribution is needed.

Factors specific to acoustic monitoring:

- Choice of detection statistic: as discussed above.
- Changes in acoustic behaviour: for click monitoring these have not emerged as problematic, but they can be assessed as changes in the distribution of click rates within click trains.
- Changes in environmental conditions: such things as a persistent increase in local noise levels, or the onset of construction work nearby require the site identity to end and would generally require a new site to be found.
- Changes in position of logger: large changes in position within the water column can substantially affect detection rates and would require a change in site identity.

Factors specific to use of PYRA:

- Large data gaps irregularly distributed: this is the issue on which PYRA generally does best, but it may effectively ‘cancel’ a large part of the data that is unpaired and give rise to the problems of few detections. It is possible that more sophisticated approaches may work better here but are particularly difficult to apply in this circumstance.

## Conclusions

Our assessment of Paired Year Ratio Assessment (PYRA) demonstrates that it is a relatively simple, and well-behaved assessment tool for multi-year point monitoring data, particularly the static acoustic data for which it was designed. It is tolerant of irregular gaps although it necessarily must fail when large gaps are both numerous and irregularly distributed in time. The level of skill and experience required to apply it, and the danger of misapplying it are less than that for the alternatives. In principle it could be applied in any context where there is a known, fixed, periodicity other than the day or the year in gappy data. In the context of static acoustic monitoring of cetaceans, PYRA is not intended to replace any specific method, but provides an additional useful tool in progressing towards trend monitoring as a cost-effective service to future conservation efforts globally.

## Supporting information

S1 Fig GAMS diagnostics plots for the synthetic data sets, harbour porpoises and orcas data. GAMS diagnostics plots are shown for the Synthetic Data sets in S3 Figures (1–4) in S1 Fig and for the harbour porpoises and orcas data in S3 Figures (5 and 6) in S1 Fig. In each Figure, the left column (top) shows a qq plot with (bottom) histogram to assess normality, the right column (top) shows residuals v fitted values to assess homogeneity, with (bottom) response v fitted values.
(DOCX)

S2 Fig PYRA and GAMs plots for synthetic data sets with high variation. S4 Figures(1–2) in S2 Fig show plots for the Scenario1 and Scenario 2 data, S4 Figure(3) in S2 Fig shows the PYRA estimator, S4 Figures (4–5) in S2 Fig show the GAMs plots and S4 Figures(6–9) in S2 Fig show the respective GAMs diagnostic plots.
(DOCX)

S3 Fig PYRA and GAMs plots for synthetic data sets with low variation. S5 Figures(1–2) in S3 Fig show plots for the Scenario1 and Scenario 2 data, S5 Figure(3) in S3 Fig shows the PYRA estimator, S5 Figures(4–5) in S3 Fig show the GAMs plots and S5 Figures(6–9) in S3 Fig show the respective GAMs diagnostic plots.
(DOCX)

S1 TablePYRA and randomisation tests for synthetic high and low variation data.Comparison of PYRA with the four randomisation trend tests applied to the synthetic data of (S6 Table 1 in S1 Table) Scenario 1 and (S6 Table 2 in S1 Table) Scenario 2 either with High or Low variation. The sample statistic *Stat* was determined by the randomisation test and is shown with p-values for each data set. RT1: Linear regression with the regression slope coefficient *m* as the sample statistic; RT2: Number of runs above or below the median value of the data; RT3: Number of runs of positive or negative differences; RT4: Count of positive or negative differences between consecutive data points. The PYRA trend statistic $\hat{P_{T}}$ with 95% percentile confidence limits (lower, upper). 5000 randomisations were used to generate the randomisation distributions and PYRA percentile confidence intervals throughout.(DOCX)

S1 Appendix Calculation of regional PYRA from multiple acoustic site data. (DOCX)

S2 Appendix GAMS plots for the Synthetic *incomplete data* of Scenario 1 and Scenario 2. S2 Figure 1 shows smoothers (solid line); s_1_ for year and s_2_ for day within the year (Yday), with 95% confidence bands (dashed) and partial residuals (dots) obtained by fitting the GAM model to the *incomplete data* of (A-B) Scenario 1 and bottom row (C-D) Scenario 2.
(DOCX)

S1 Data (ZIP)
