# Supplementary material for: Estimating cetacean population trends from static acoustic monitoring data using Paired Year Ratio Assessment (PYRA)
Source: PLoS One. 2022 Mar 17;17(3):e0264289. doi: 10.1371/journal.pone.0264289 (PMC8929582; doi:10.1371/journal.pone.0264289)
Supplement: S1 Fig — GAMS diagnostics plots are shown for the Synthetic Data sets in S3 Figures (1–4) in S1 Fig and for the harbour porpoises and orcas data in S3 Figures (5 and 6) in S1 Fig. In each Figure, the left column (top) shows a qq plot with (bottom) histogram to assess normality, the right column (top) shows residuals v fitted values to assess homogeneity, with (bottom) response v fitted values. (DOCX) [file pone.0264289.s001.docx]

**S3 Figures 1-6. GAMs Diagnostics plots for the Synthetic Data sets, harbour porpoises and orcas data.** GAMs diagnostics plots are shown for the Synthetic Data sets in S3 Figures(1-4) and for the harbour porpoises and orcas data in S3 Figures (5 and 6). In each Figure, the left column (top) shows a qq plot with (bottom) histogram to assess normality, the right column (top) shows residuals v fitted values to assess homogeneity, with (bottom) response v fitted values.

**
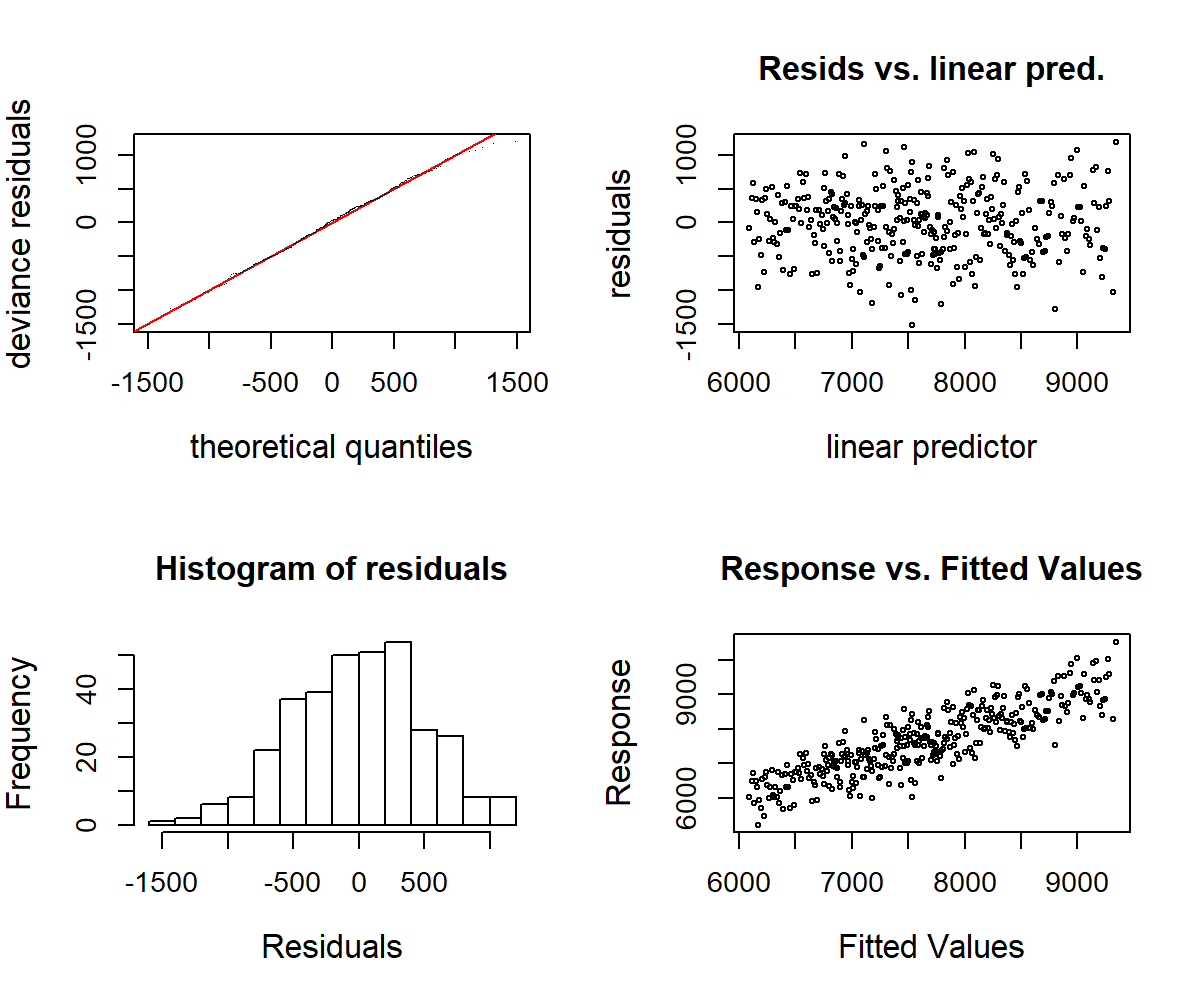
**

**S3 Figure 1. Scenario 1 paired data (reasonable).**

(Left top and bottom) show that the normal distributive assumption is reasonable; (Right top) shows residuals display some heterogeneity, against the assumption of constant variance; (Right bottom) shows that model predictive ability is fair.


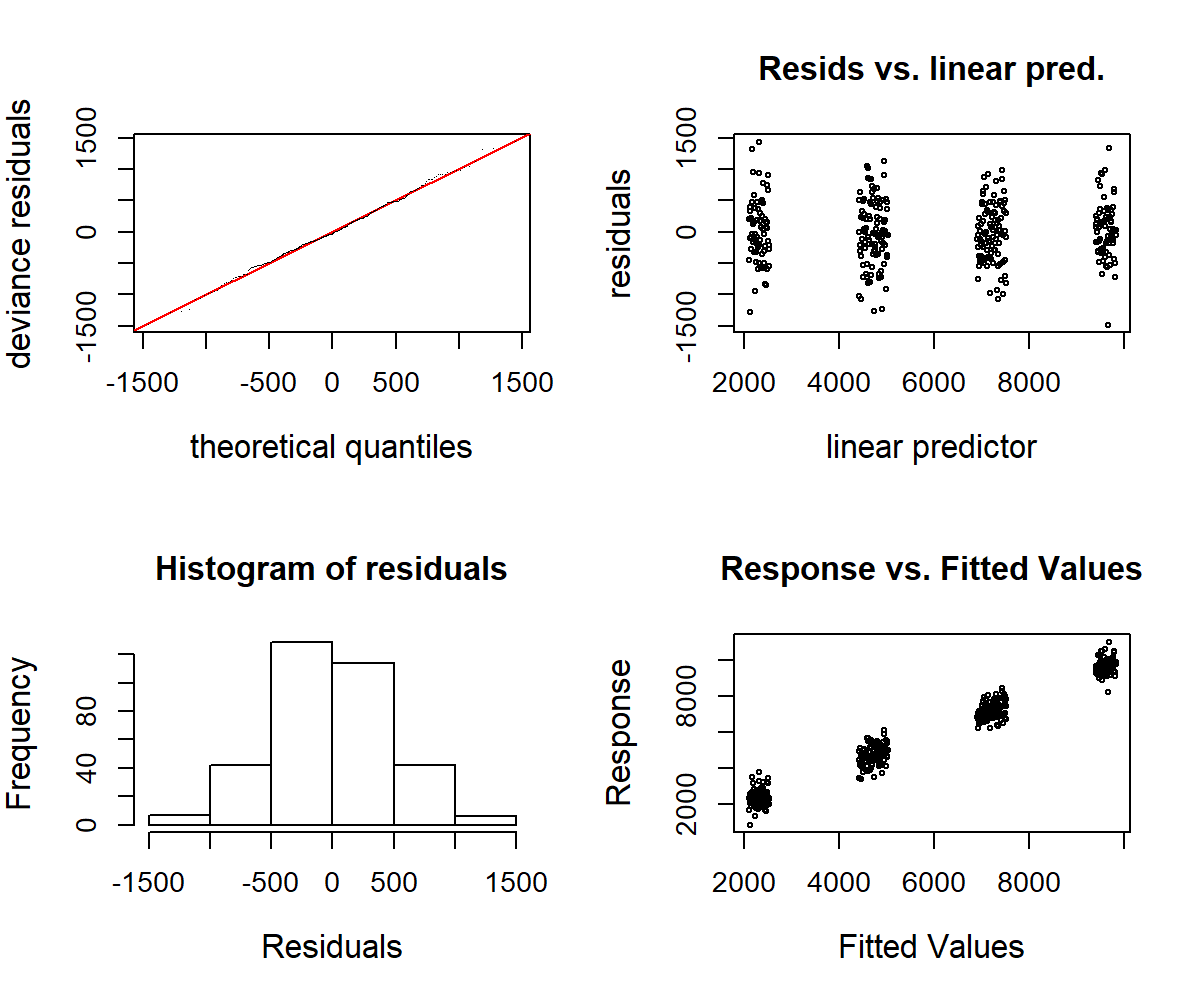


**S3 Figure 2. Scenario 2 paired data (reasonable).**

(Left top and bottom) show that the normal distributive assumption is reasonable; (Right top) shows residuals display some heterogeneity, against the assumption of constant variance; (Right bottom) shows that model predictive ability is fair.


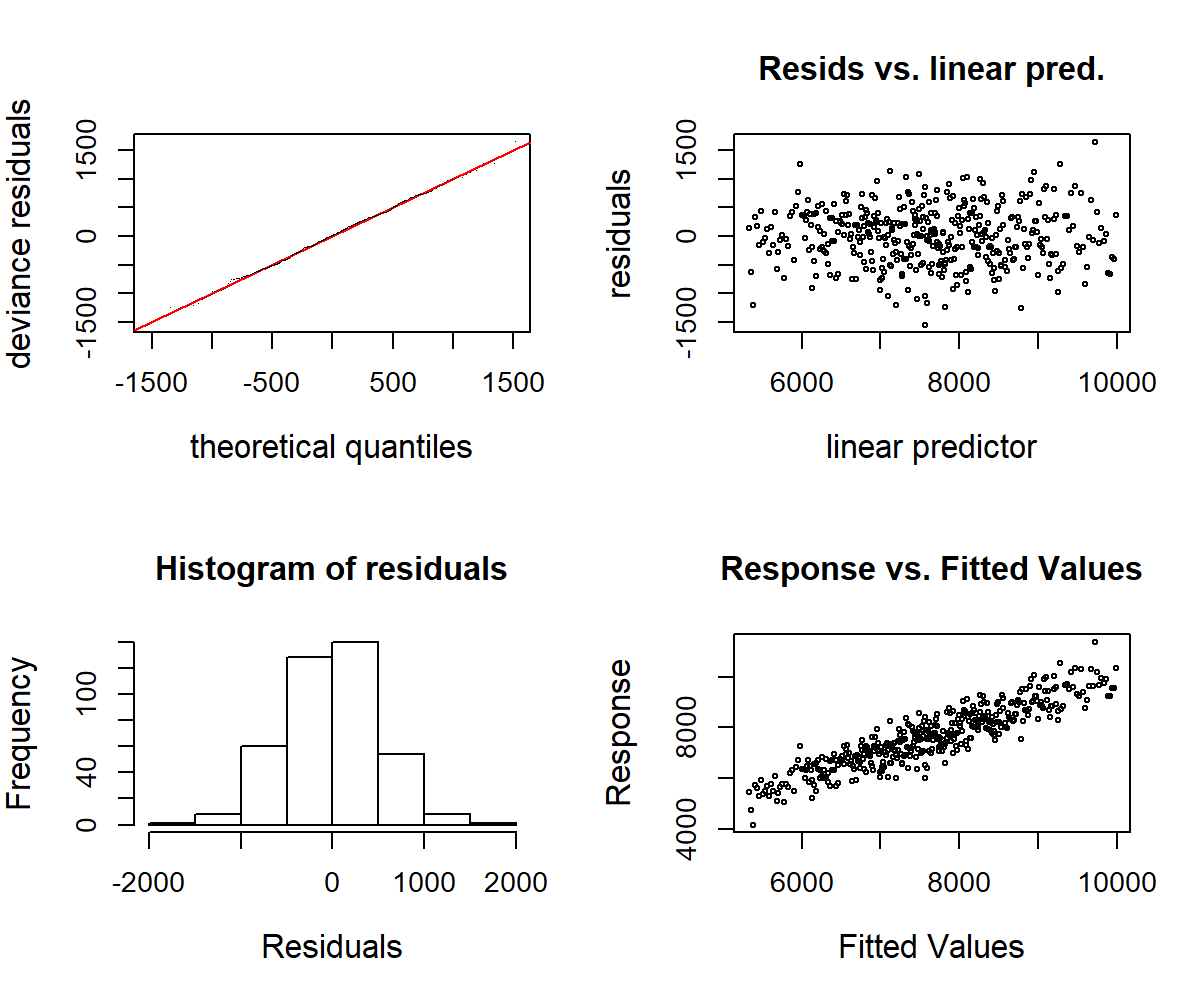


**S3 Figure 3. Scenario 1 incomplete data (good)**

(Left top and bottom) show that the normal distributive assumption is reasonable; (Right top) shows residuals display reasonable homogeneity, in line with a constant variance; (Right bottom) shows that model predictive ability is good.


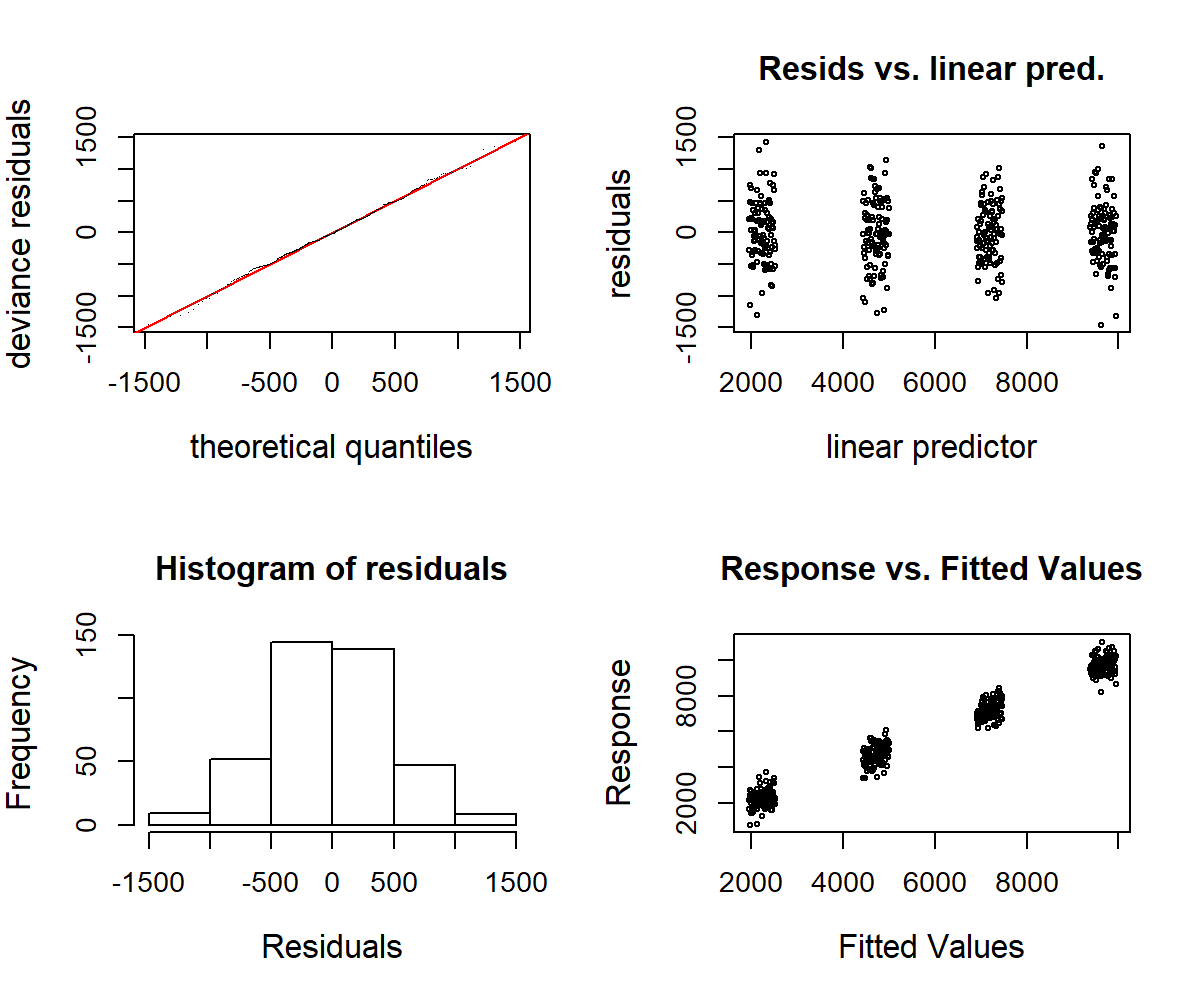


**S3 Figure 4. Scenario 2 incomplete data (good).**

(Left top and bottom) show that the normal distributive assumption is reasonable; (Right top) shows residuals display reasonable homogeneity, in line with a constant variance; (Right bottom) shows that model predictive ability is good.


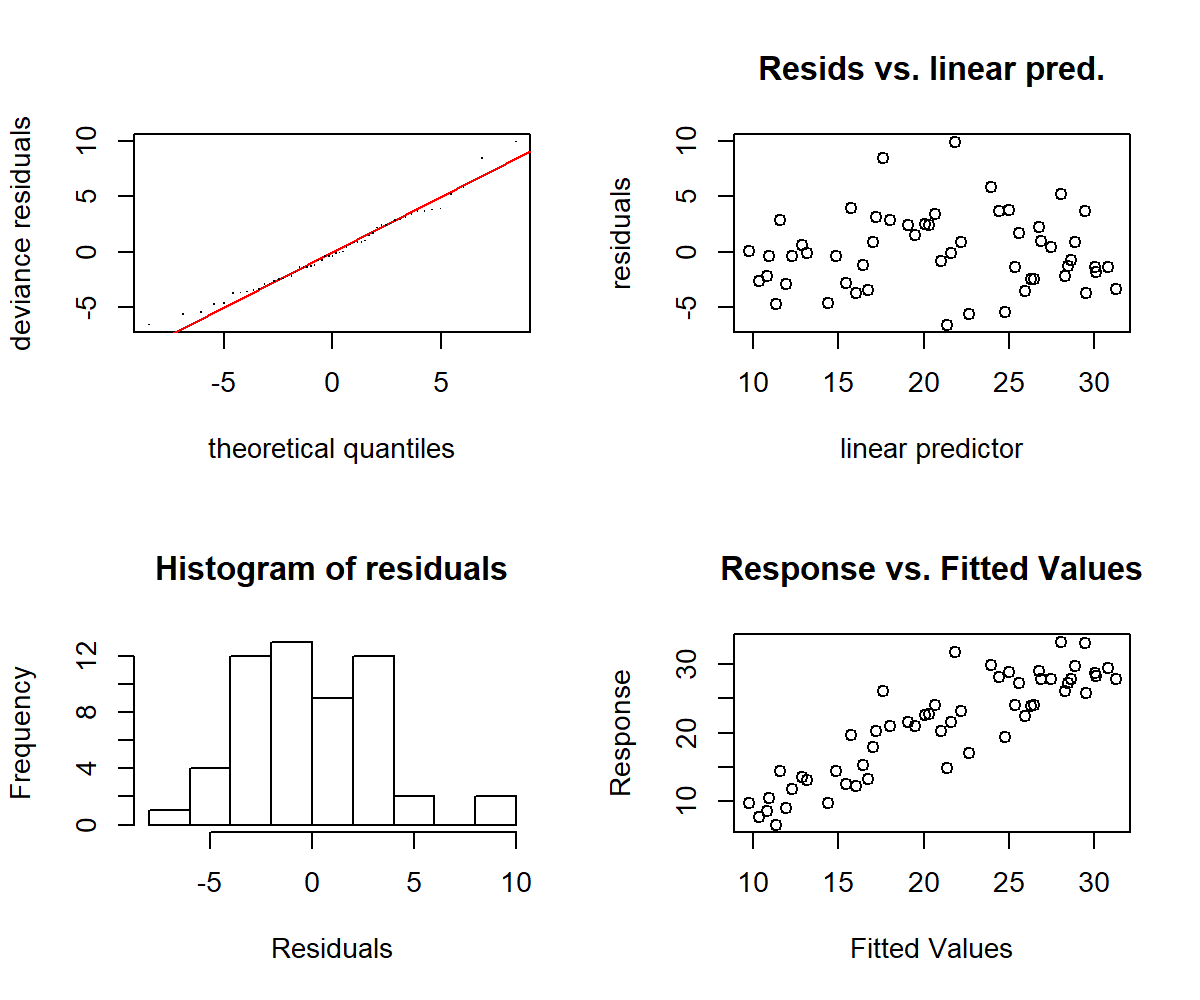


**S3 Figure 5. Harbour porpoises (reasonable).**

(Left top and bottom) show that the normal distributive assumption is weak; (Right top) shows residuals display some heterogeneity, against the assumption of constant variance; (Right bottom) shows that model predictive ability is fair.


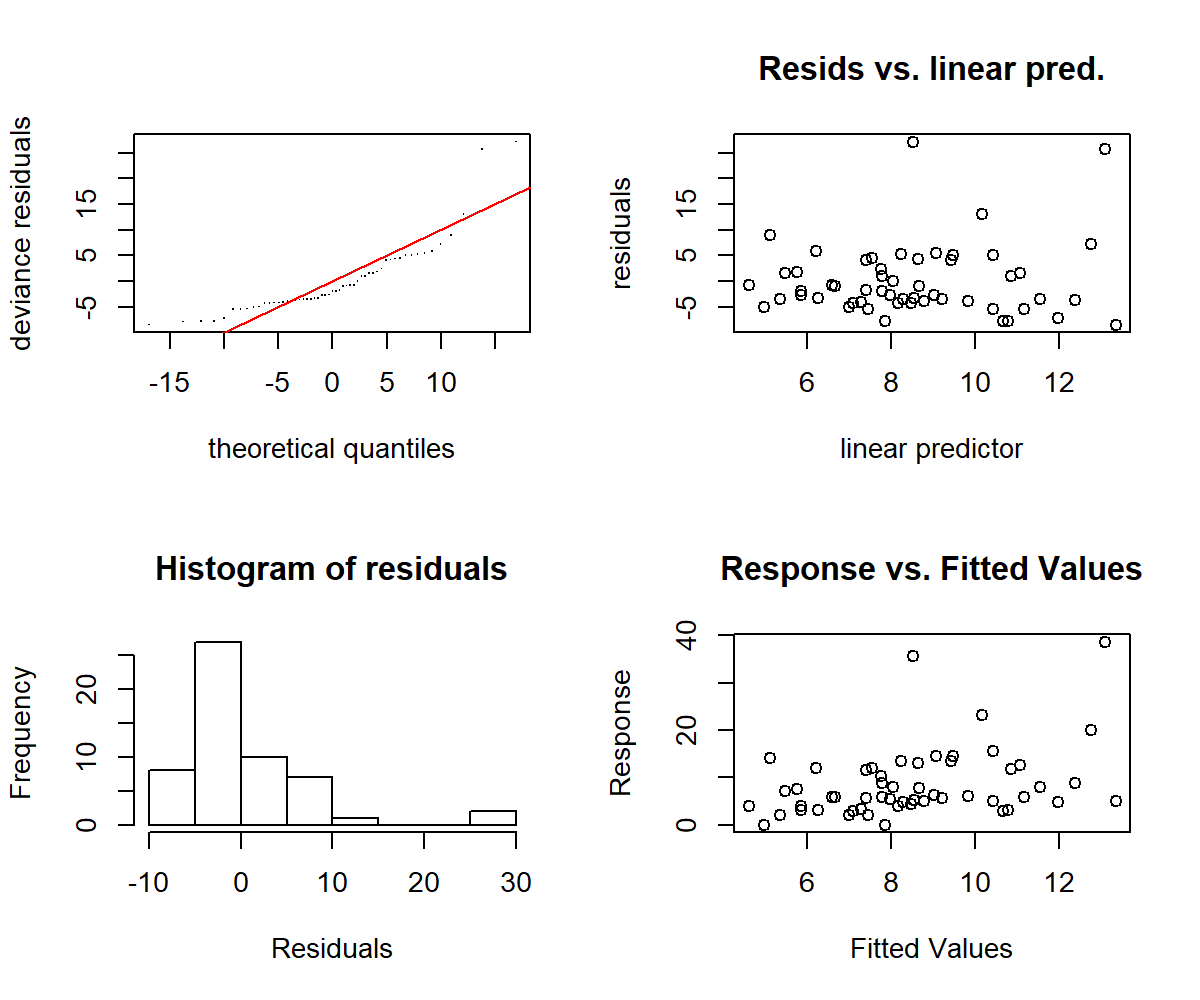


**S3 Figure 6. Orcas (weak).**

(Left top and bottom) show that the normal distributive assumption is contravened; (Right top) shows residuals display heterogeneity, contravening the assumption of constant variance; (Right bottom) shows that model predictive ability is poor.
