# Supplementary material for: Estimating cetacean population trends from static acoustic monitoring data using Paired Year Ratio Assessment (PYRA)
Source: PLoS One. 2022 Mar 17;17(3):e0264289. doi: 10.1371/journal.pone.0264289 (PMC8929582; doi:10.1371/journal.pone.0264289)
Supplement: S2 Fig — S4 Figures(1–2) in S2 Fig show plots for the Scenario1 and Scenario 2 data, S4 Figure(3) in S2 Fig shows the PYRA estimator, S4 Figures (4–5) in S2 Fig show the GAMs plots and S4 Figures(6–9) in S2 Fig show the respective GAMs diagnostic plots. (DOCX) [file pone.0264289.s002.docx]

**S4 Figures 1-9. PYRA and GAMs plots for Synthetic data sets with High Variation.** S4 Figures(1-2) show plots for the Scenario1 and Scenario 2 data, S4 Figure(3) shows the PYRA estimator, S4 Figures (4-5) show the GAMs plots and S4 Figures(6-9) show the respective GAMs diagnostic plots.


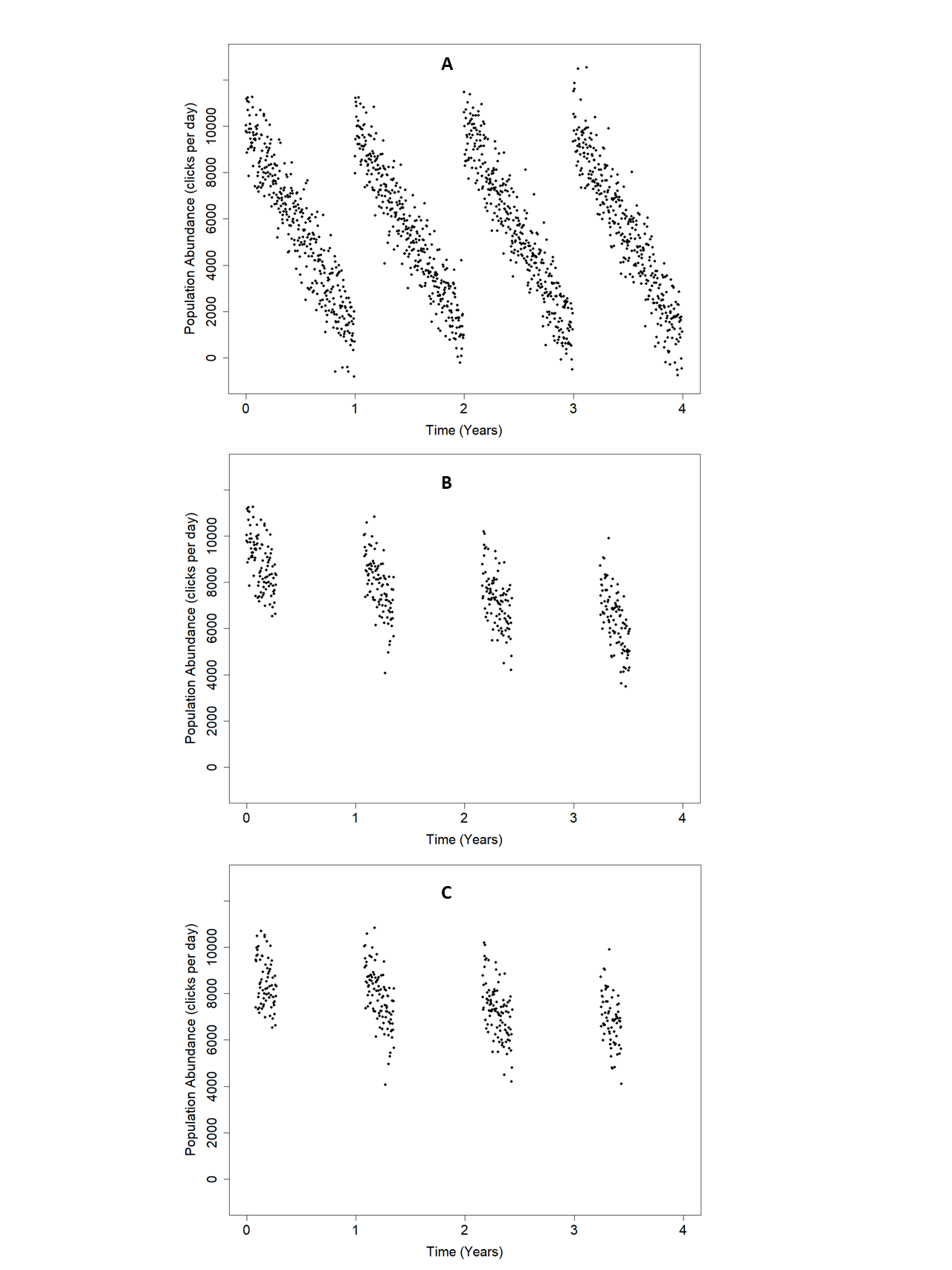


**S4 Figure 1.** The High Variation Scenario 1 periodic population pattern with no trend, plotted as time series for the 3 data subsets. (A) *complete data*, (B) *incomplete data* and (C) *paired data*. The apparent downward trend visible in (B) and (C) is spurious.

**
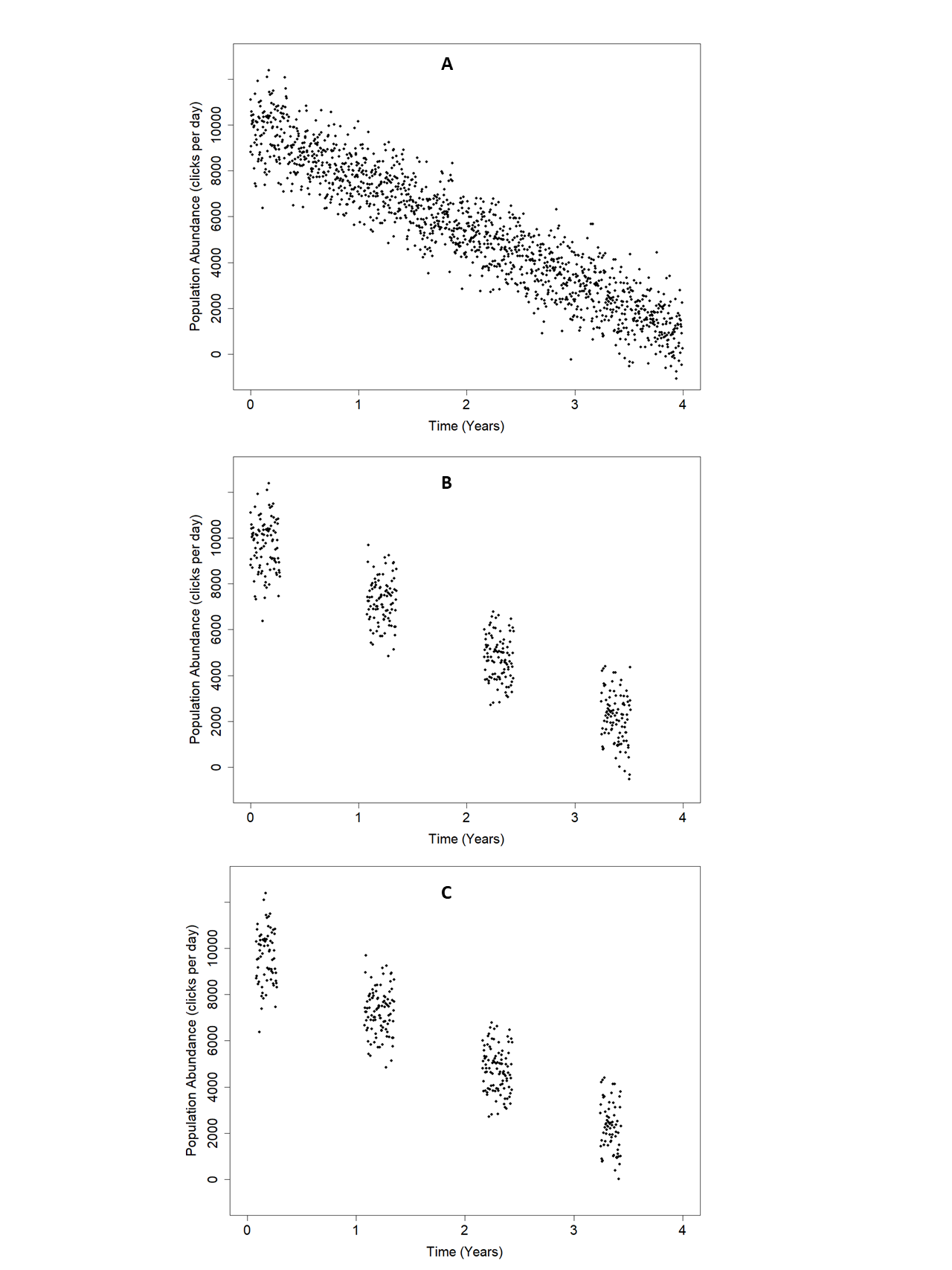
**

**S4 Figure 2.** The High Variation Scenario 2 downward population trend shown plotted as time series for the 3 data subsets. (A) *complete data*, (B) *incomplete data* and (C) *paired data*.


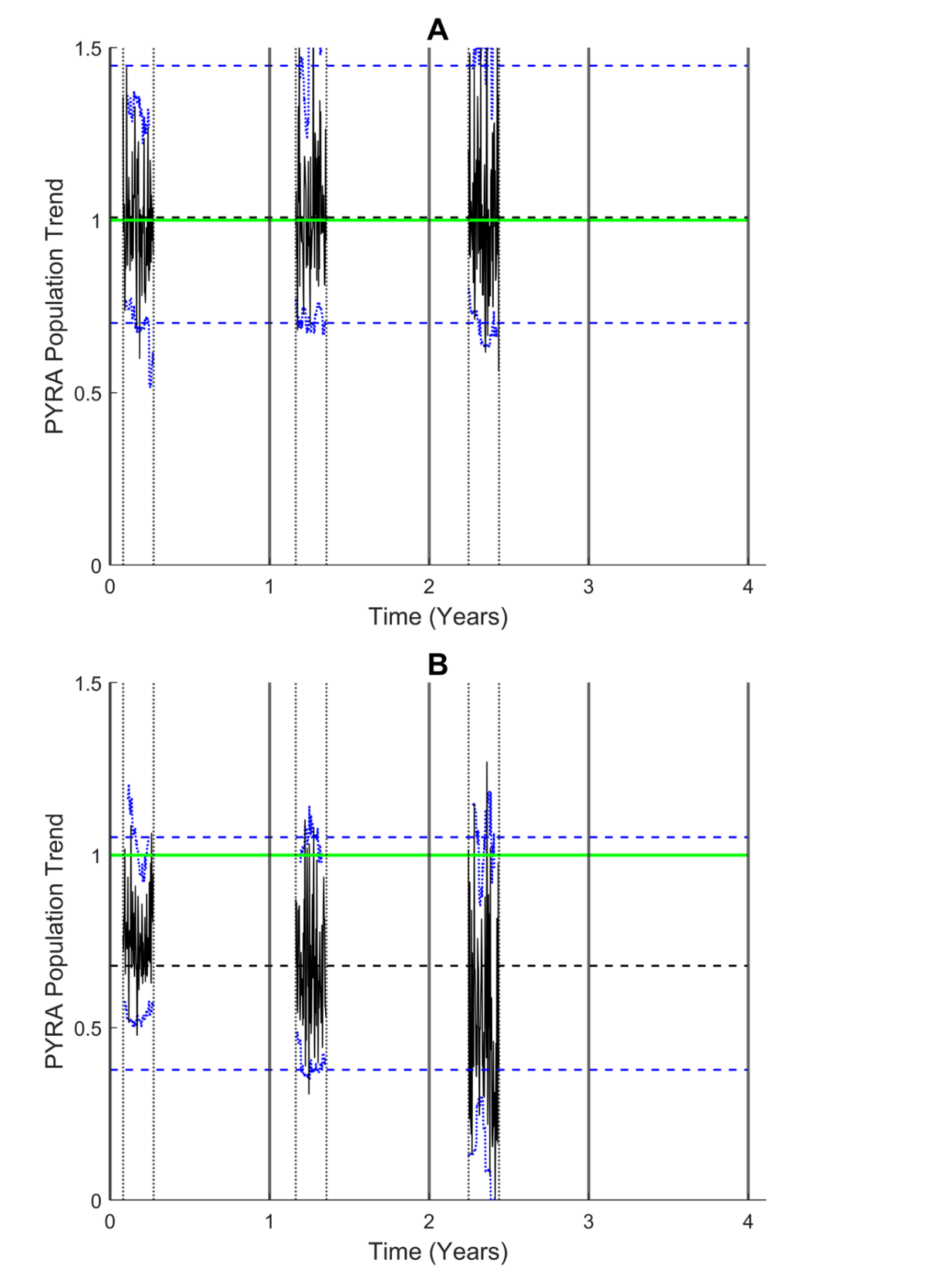


**S4 Figure 3.** The PYRA statistical estimator $\hat{P}(t^{*})$ plotted (solid black) for the High Variation *paired data sets* against time over the time span T of the data set with 95% percentile lower (L) and upper (U) confidence limits (dotted blue) shown for (A) Scenario 1 and (B) Scenario 2. The respective mean averages of$\tilde{\left[ \hat{P}\left( t^{*} \right) \right]=}\hat{P}_{T}$ are superimposed (dashed black) together with 95% percentile confidence limits (dashed blue). The baseline ‘no trend’ PYRA of 1 is the horizontal green line. In (A) the PYRA trend statistic$\hat{P_{T}}$= 0.995 (L=0.932, U=1.003) indicates the trend is flat; in (B)$\hat{P_{T}}$= 0.666 (L=0.629, U=0.688) indicates a downward trend, estimated at 33% [(1-0.666) x 100] over the time span T of the data set.


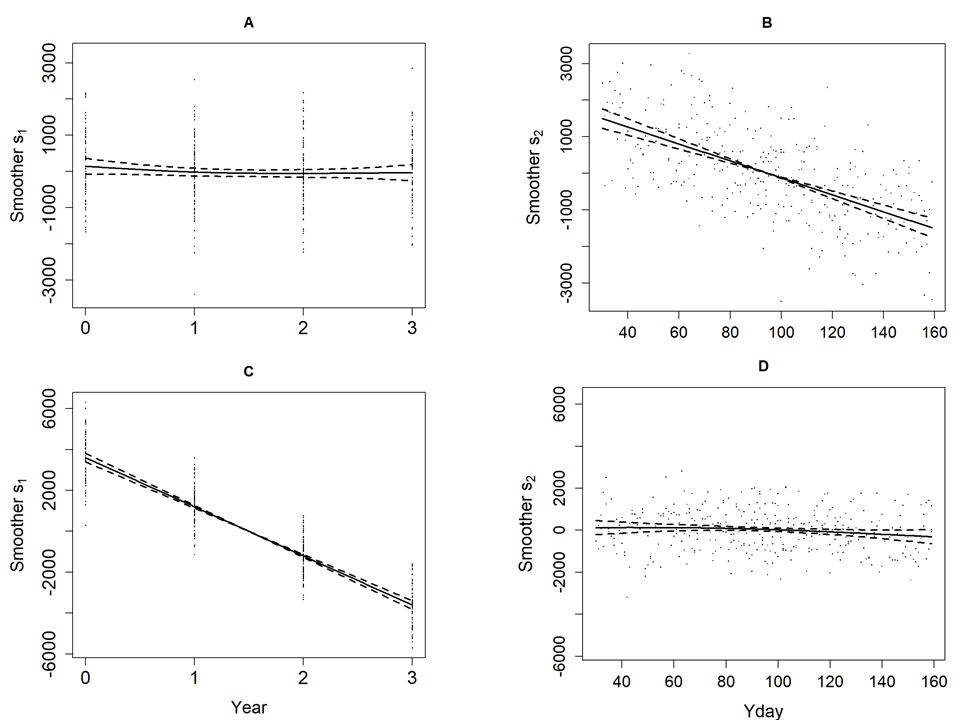


**S4 Figure 4.** Smoothers (solid) s1 for Year and s2 for Yday, with 95% confidence bands (dashed) and partial residuals (dots) obtained by fitting the GAM model to the High Variation *paired data* of (A-B) Scenario 1 and bottom row (C-D) Scenario 2. For Scenario 1, these respectively indicate (A) no long-term trend (p=0.379); (B) the seasonal decline pattern is highly statistically significant (p= 2e-16). Conversely, for Scenario 2, (C) the long-term decline trend is highly statistically significant (p= 2e-16); (D) the seasonal linear downward trend is not statistically significant (p= 0.153). The fitted GAM model diagnostics were reasonable for both Scenarios (see S4 Figures (6-7)).

**
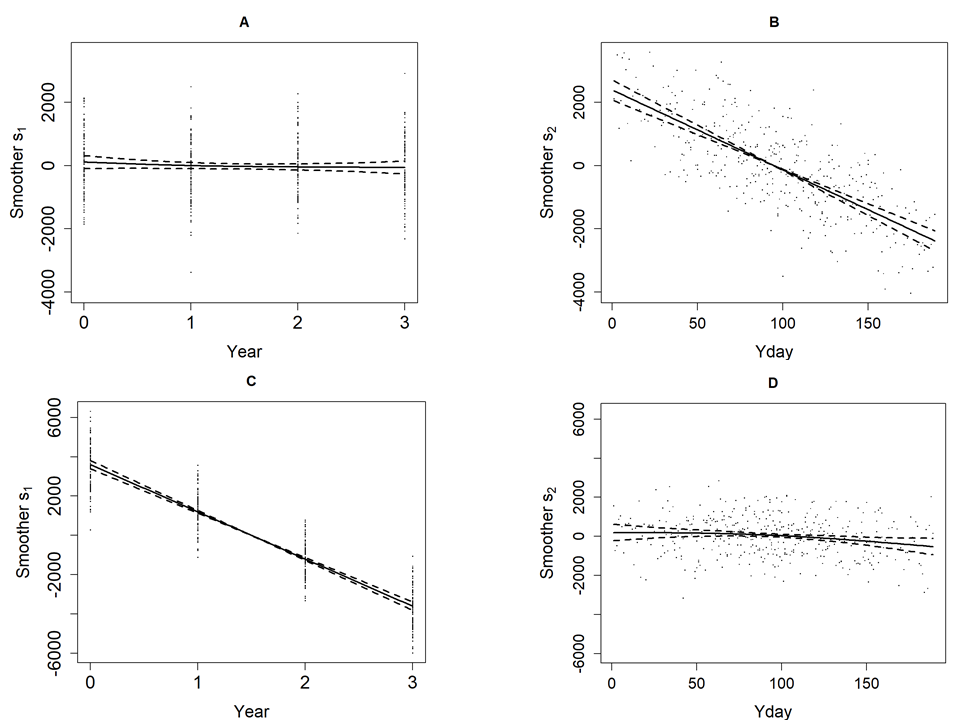
**

**S4 Figure 5.** Smoothers (solid) s1 for Year and s2 for Yday, with 95% confidence bands (dashed) and partial residuals (dots) obtained by fitting the GAM model to the High Variation *incomplete data* of (A-B) Scenario 1 and bottom row (C-D) Scenario 2. For Scenario 1, these respectively indicate (A) no statistically significant long-term trend (p=0.436); (B) a seasonal decline pattern which is highly statistically significant (p= 2e-16). Conversely, for Scenario 2, (C) the long-term decline trend is highly statistically significant (p= 2e-16); (D) the seasonal linear downward trend is not statistically significant (p= 0.0457). The fitted GAM model diagnostics were reasonable for both Scenarios (see S4 Figures (8-9).

**
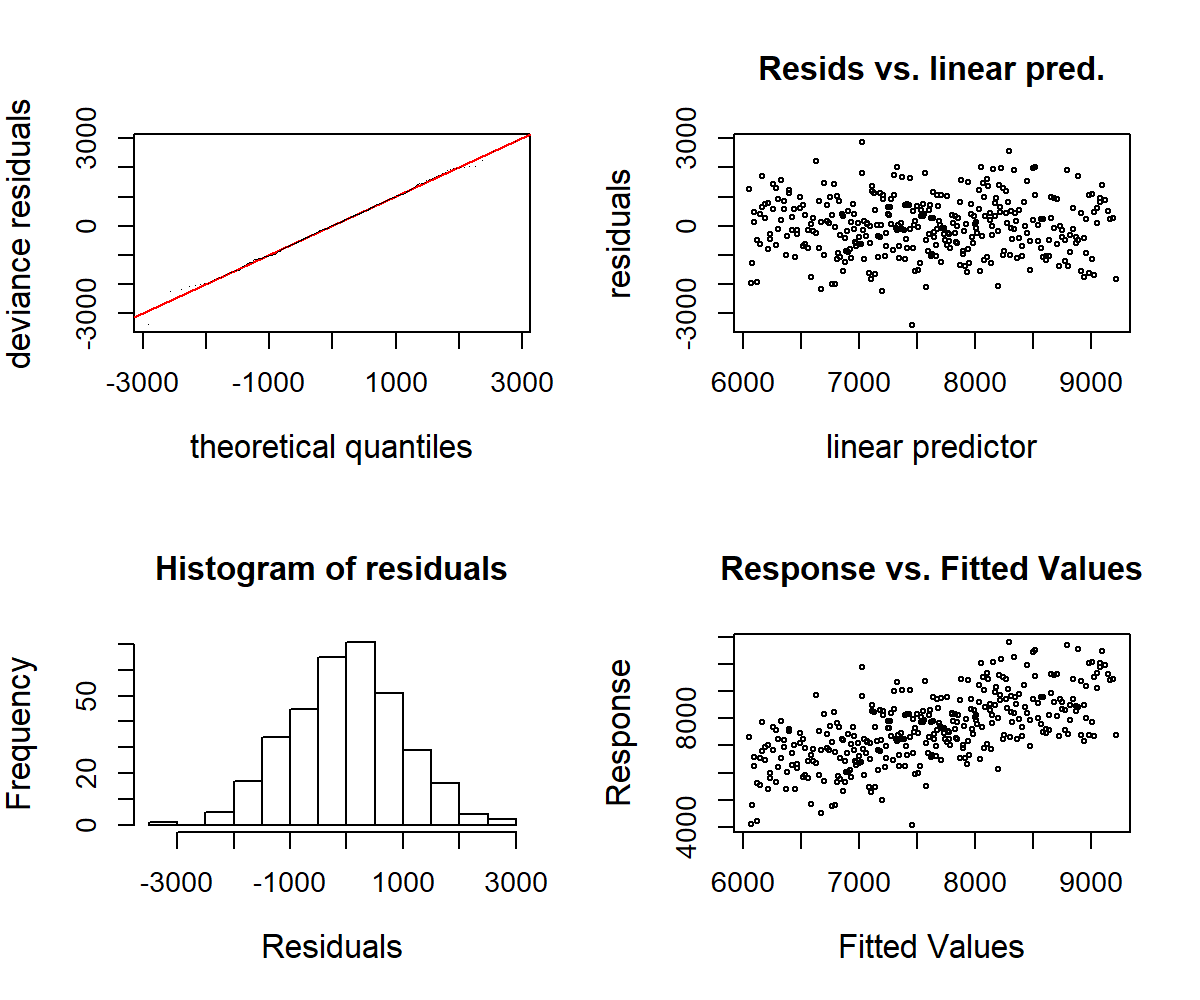
**

**S4 Figure 6.** Diagnostics plots for the GAM model shown in S4 Figure 4(AB) fitted to the Scenario 1 High Variation *paired data*. The left column (top) shows a qq plot with (bottom) histogram to assess normality, the right column (top) shows residuals v fitted values to assess homogeneity, with (bottom) response v fitted values.

**
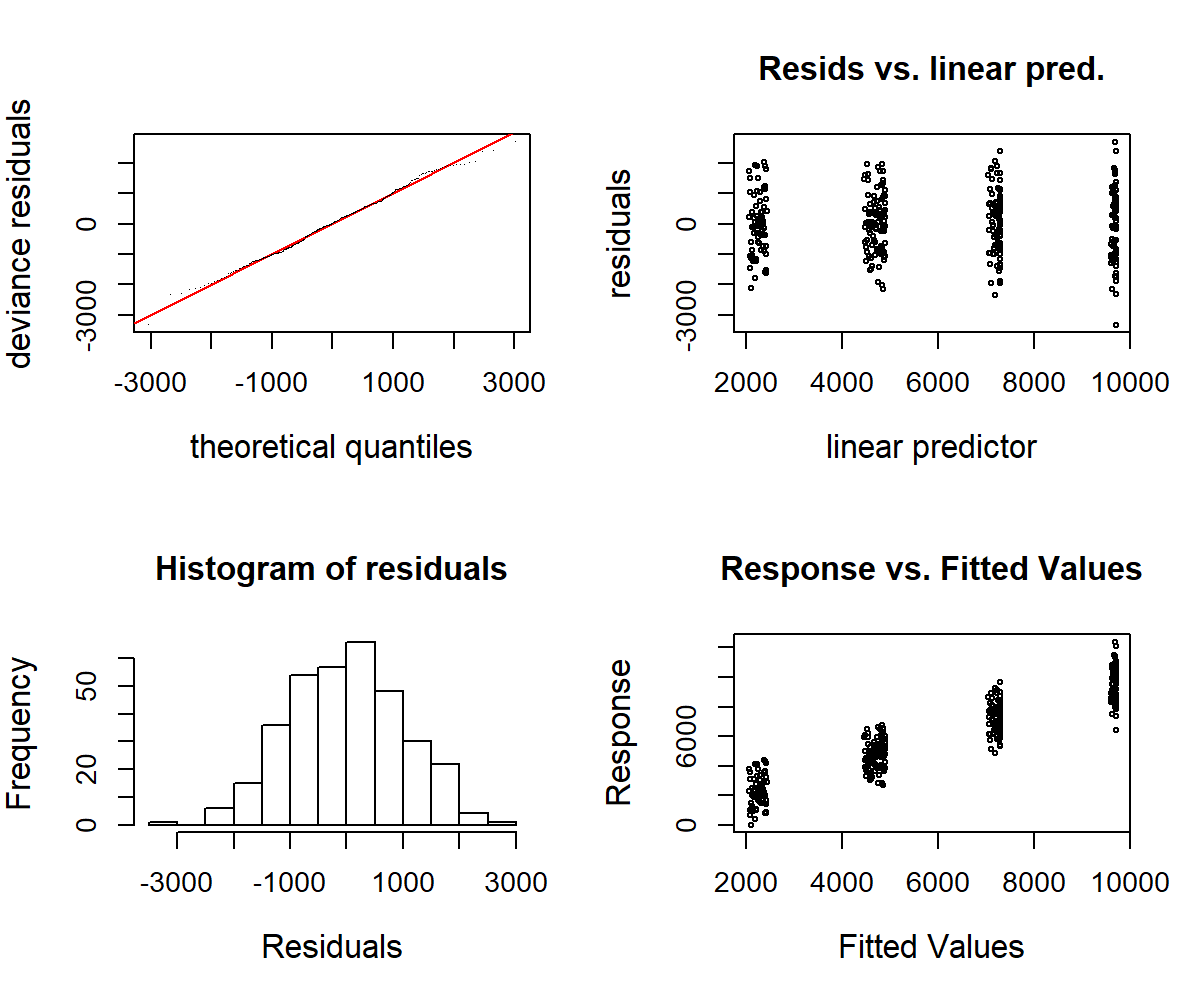
**

**S4 Figure 7.** Diagnostics plots for the GAM model of S4 Figure 4(CD) fitted to the Scenario 2 High Variation *paired data*. The left column (top) shows a qq plot with (bottom) histogram to assess normality, the right column (top) shows residuals v fitted values to assess homogeneity, with (bottom) response v fitted values.

**
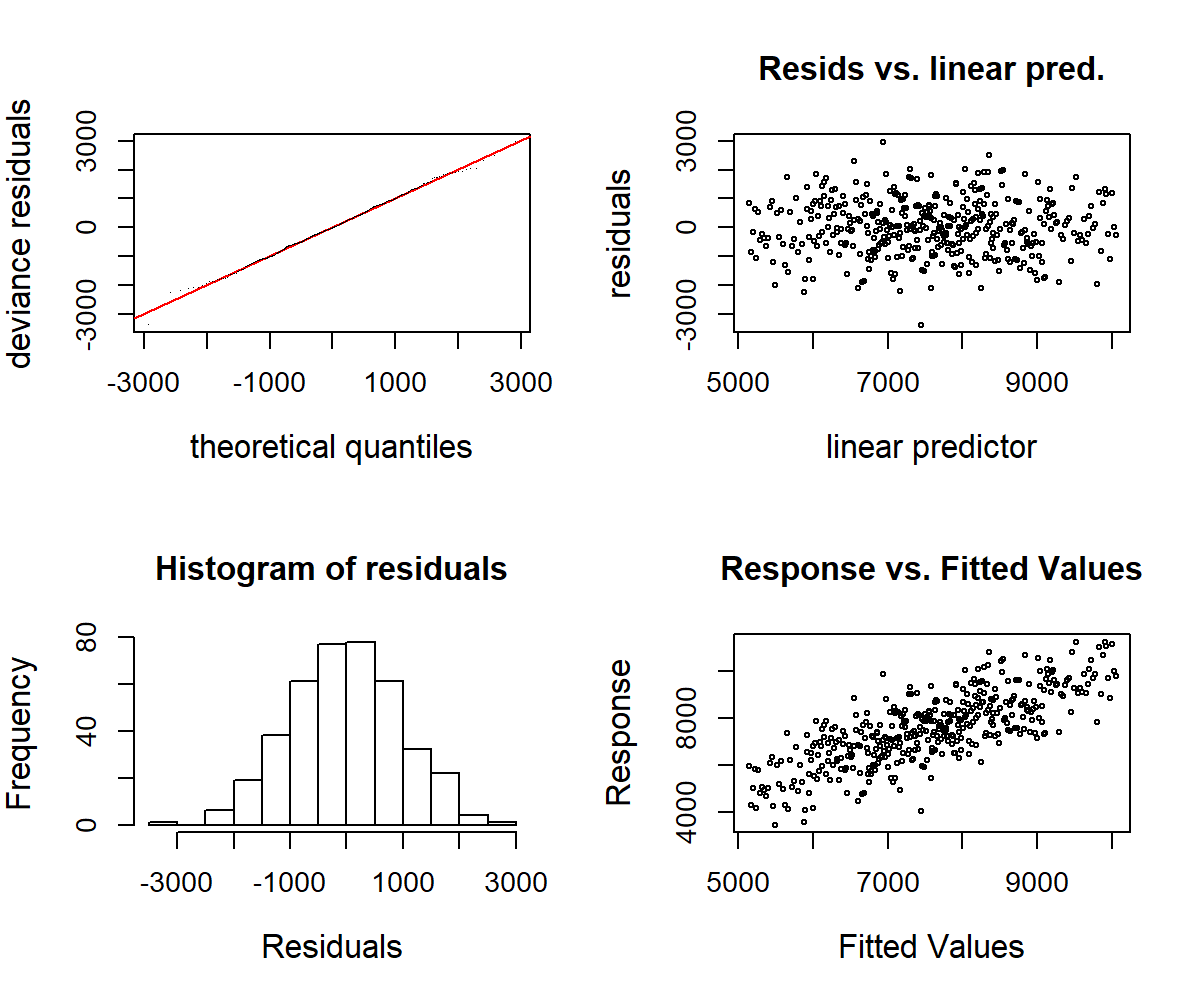
**

**S4 Figure 8.** Diagnostics plots for the GAM model of S4 Figure 5(AB) fitted to the of Scenario 1 High Variation *incomplete data*. The left column (top) shows a qq plot with (bottom) histogram to assess normality, the right column (top) shows residuals v fitted values to assess homogeneity, with (bottom) response v fitted values.

**
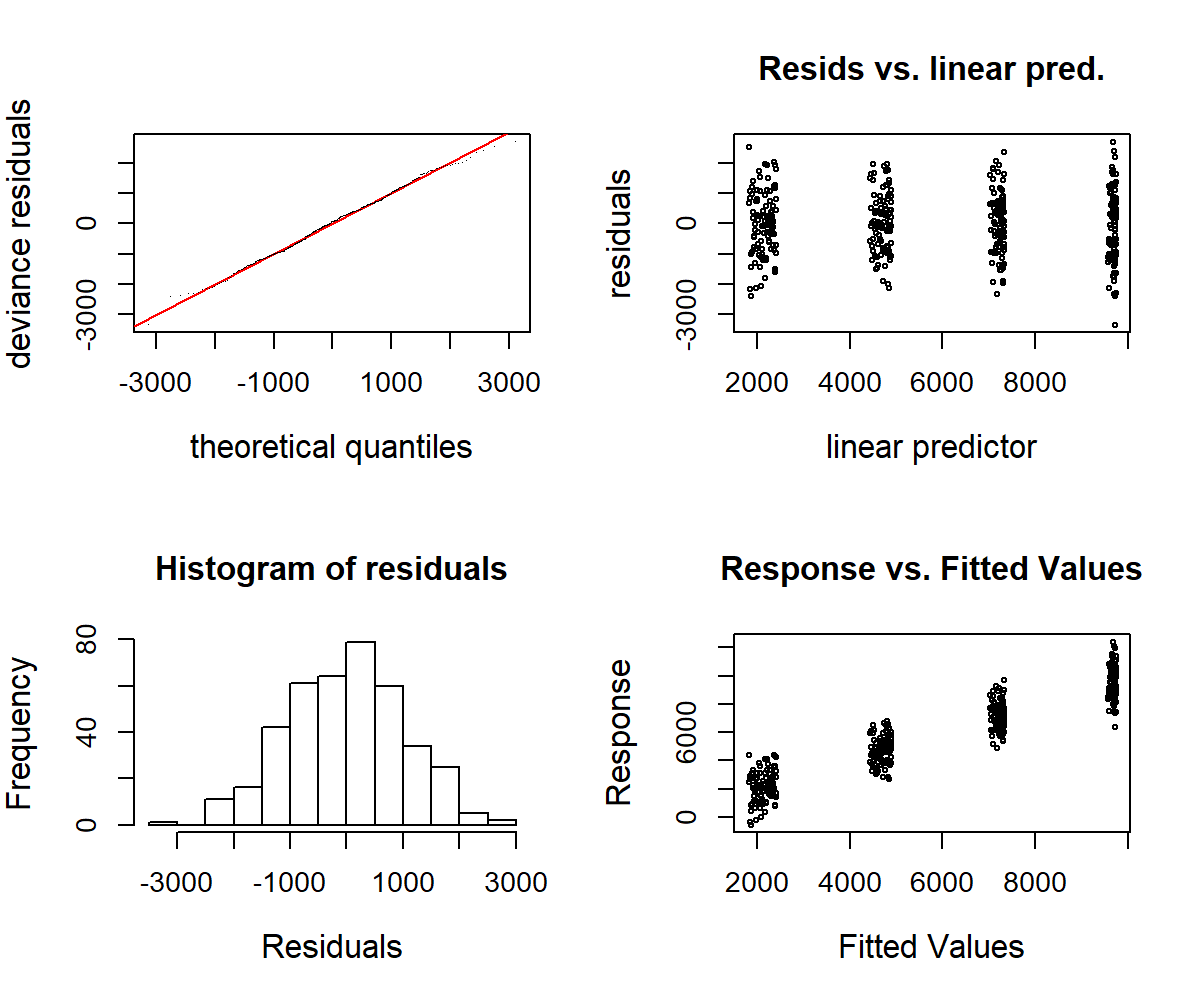
**

**S4 Figure 9.** Diagnostics plots for the GAM model of S4 Figure 5(CD) fitted to the Scenario 2 High Variation *incomplete data* . The left column (top) shows a qq plot with (bottom) histogram to assess normality, the right column (top) shows residuals v fitted values to assess homogeneity, with (bottom) response v fitted values.
