# Supplementary material for: Estimating cetacean population trends from static acoustic monitoring data using Paired Year Ratio Assessment (PYRA)
Source: PLoS One. 2022 Mar 17;17(3):e0264289. doi: 10.1371/journal.pone.0264289 (PMC8929582; doi:10.1371/journal.pone.0264289)
Supplement: S3 Fig — S5 Figures(1–2) in S3 Fig show plots for the Scenario1 and Scenario 2 data, S5 Figure(3) in S3 Fig shows the PYRA estimator, S5 Figures(4–5) in S3 Fig show the GAMs plots and S5 Figures(6–9) in S3 Fig show the respective GAMs diagnostic plots. (DOCX) [file pone.0264289.s003.docx]

**S5 Figures 1-9. PYRA and GAMs plots for Synthetic Data sets with Low Variation.** S5 Figures(1-2) show plots for the Scenario1 and Scenario 2 data, S5 Figure(3) shows the PYRA estimator, S5 Figures(4-5) show the GAMs plots and S5 Figures(6-9) show the respective GAMs diagnostic plots.


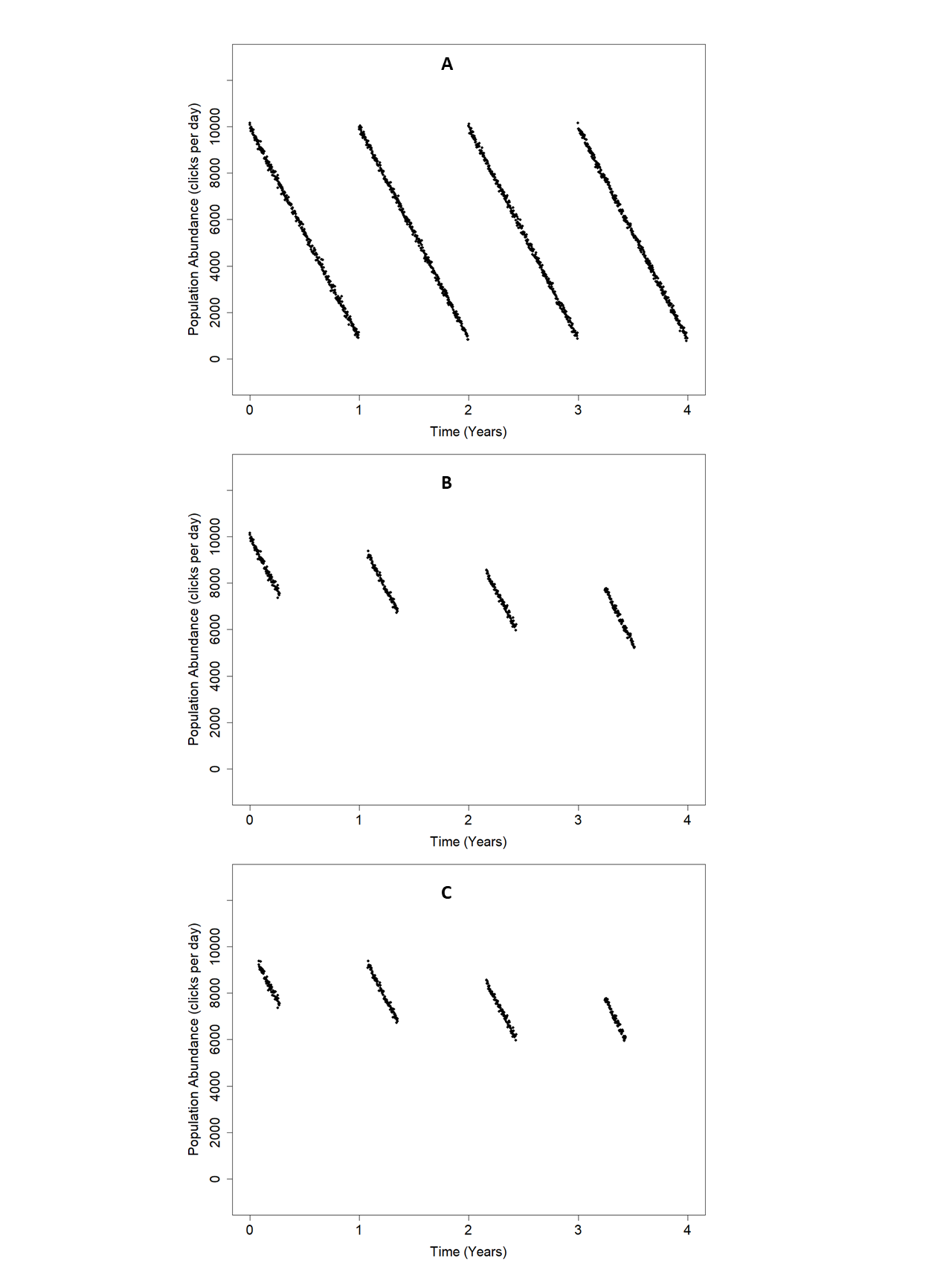


**S5 Figure 1.** The Low Variation Scenario 1 periodic population pattern with no trend, plotted as time series for the 3 data subsets. (A) *complete data*, (B) *incomplete data* and (C) *paired data*. The apparent downward trend visible in (B) and (C) is spurious.


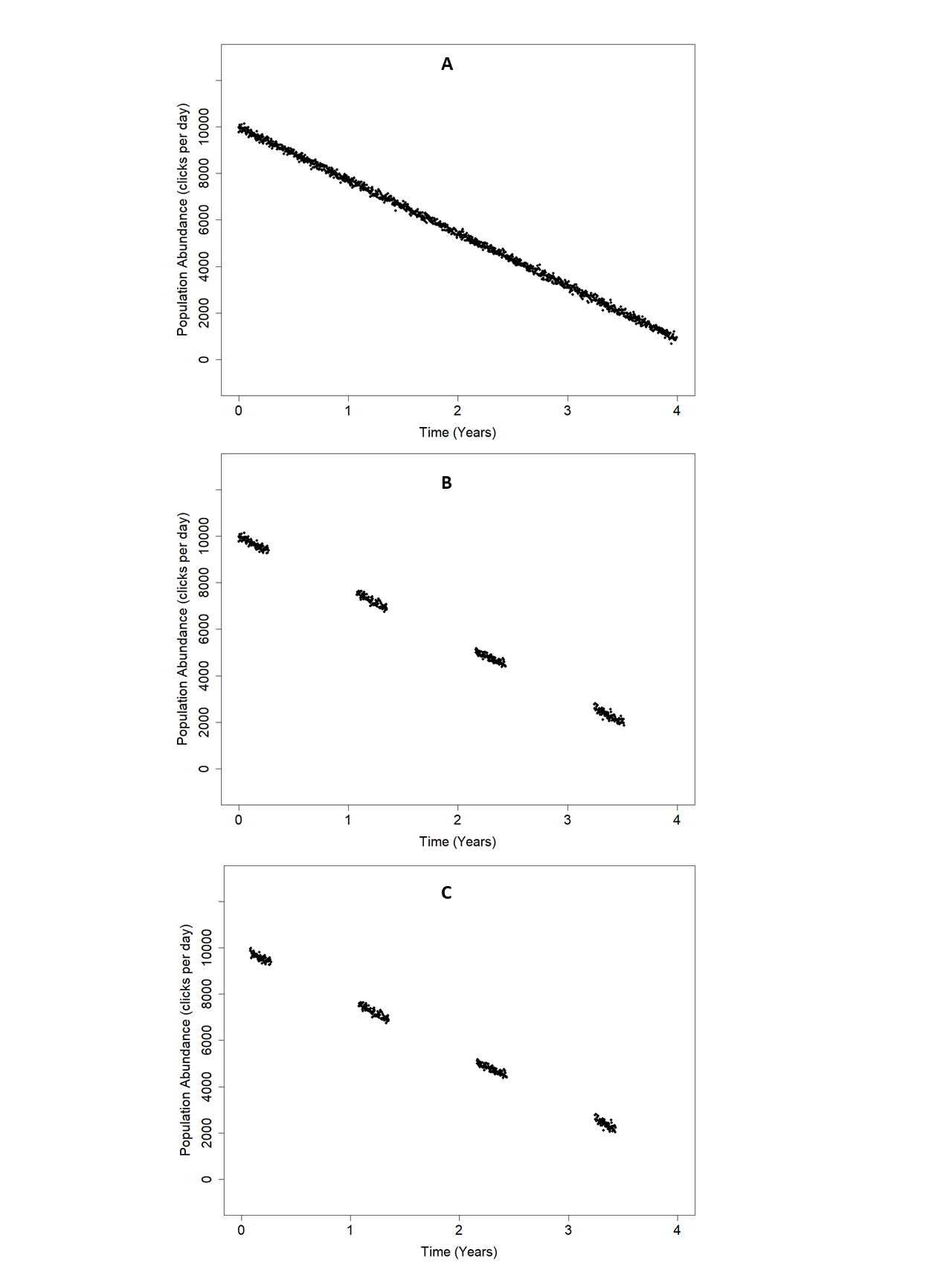


**S5 Figure 2.** The Low Variation Scenario 2 downward population trend shown plotted as time series for the 3 data subsets. (A) *complete data*, (B) *incomplete data* and (C) *paired data*.


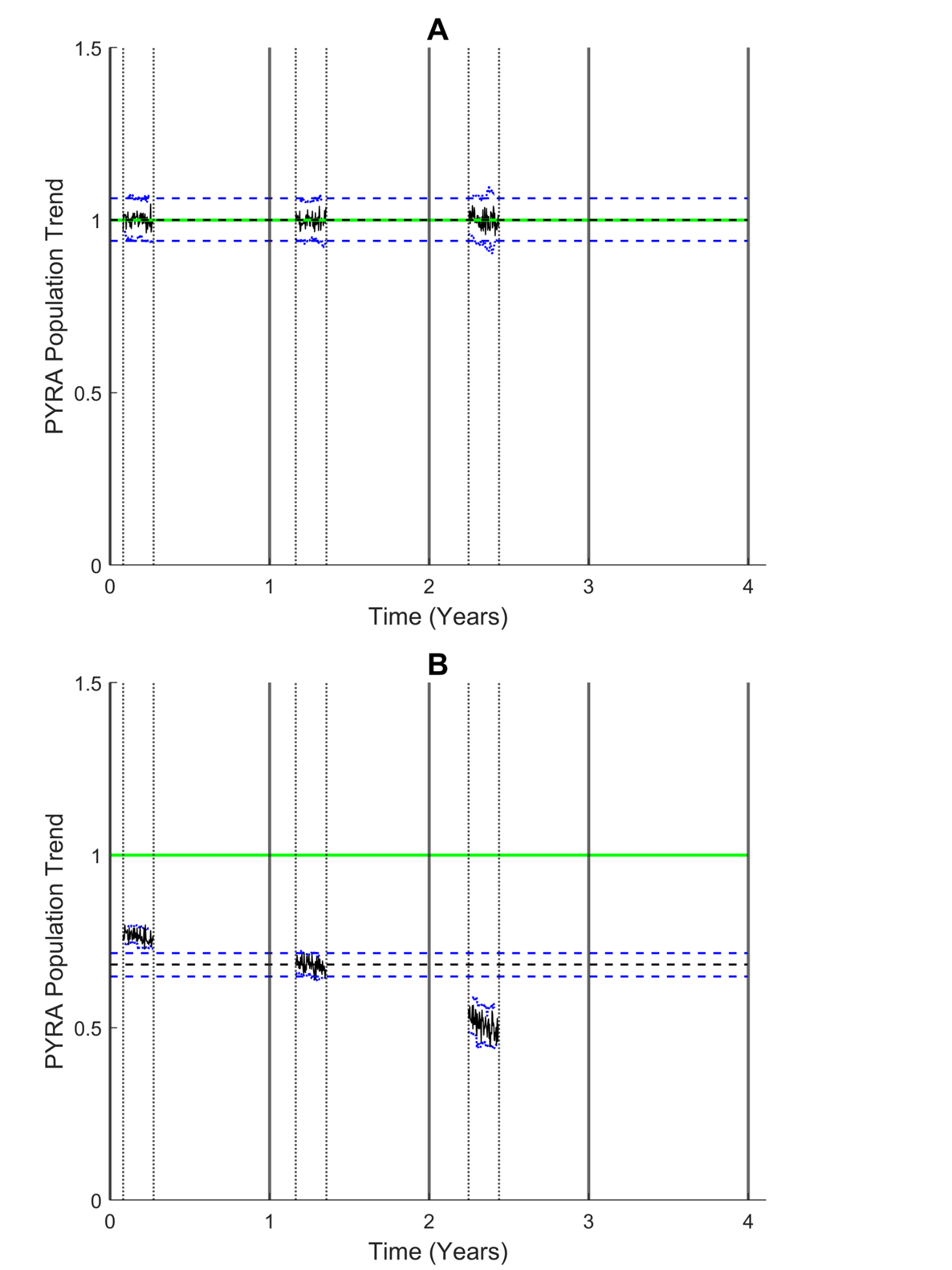


**S5 Figure 3.** The PYRA statistical estimator $\hat{P}\left( t^{*} \right)$ plotted (solid black) for the Low Variation *paired data sets* against time over the time span T of the data set with 95% percentile lower (L) and upper (U) confidence limits (dotted blue) shown for (A) Scenario 1 and (B) Scenario 2. The respective trend mean averages $\tilde{\left[ \hat{P}\left( t^{*} \right) \right]=}\hat{P}_{T}$ are superimposed (dashed black) together with 95% percentile confidence limits (dashed blue) . The baseline ‘no trend’ PYRA value of 1 is the horizontal green line. In (A) the PYRA trend statistic $\hat{P_{T}}$ = 0.999 (L=0.941, U=1.005) indicates the trend is flat; in (B)$\hat{P_{T}}$ = 0.680 (L=0.652, U=0.699) indicates a downward trend, estimated at 32% [(1-0.680) x 100] over the time span T of the data set.

**
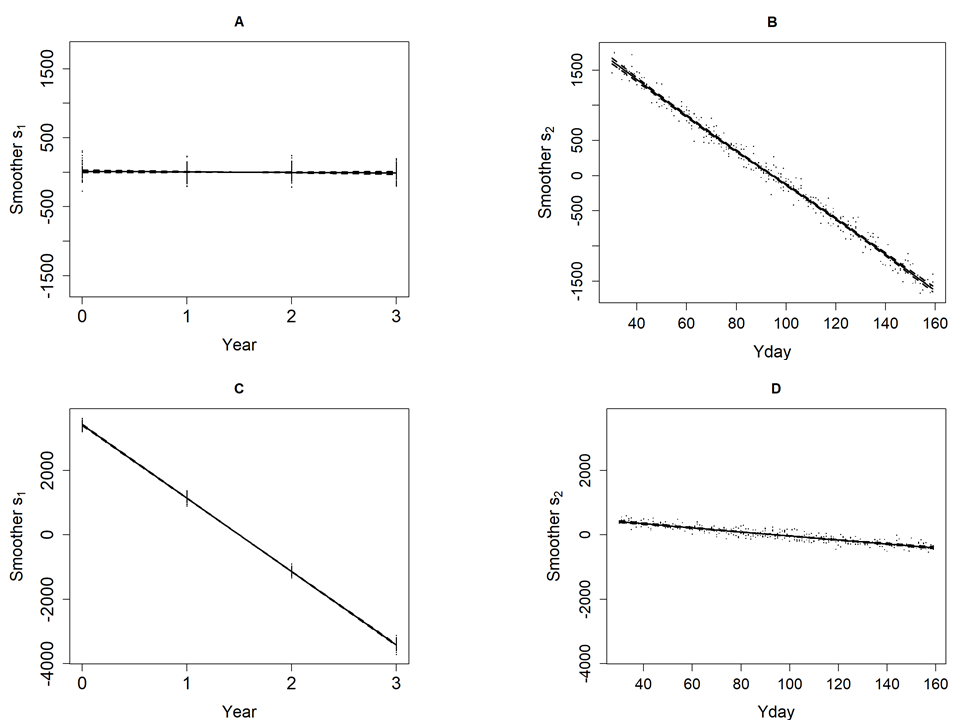
**

**S5 Figure 4.** Smoothers (solid) s1 for Year and s2 for Yday, with 95% confidence bands (dashed) and partial residuals (dots) obtained by fitting the GAM model to the Low Variation *paired data* of (A-B) Scenario 1 and bottom row (C-D) Scenario 2. For Scenario 1, these respectively indicate (A) no statistically significant long-term trend (p=0.263); (B) the seasonal decline is highly statistically significant (p= 2e-16). Conversely, for Scenario 2, (C) the long-term downward trend is highly statistically significant (p= 2e-16); (D) the seasonal linear downward trend is highly statistically significant (p= 2e-16). The fitted GAM model diagnostics were reasonable for both Scenarios (see S5 Figures (6-7)).

**
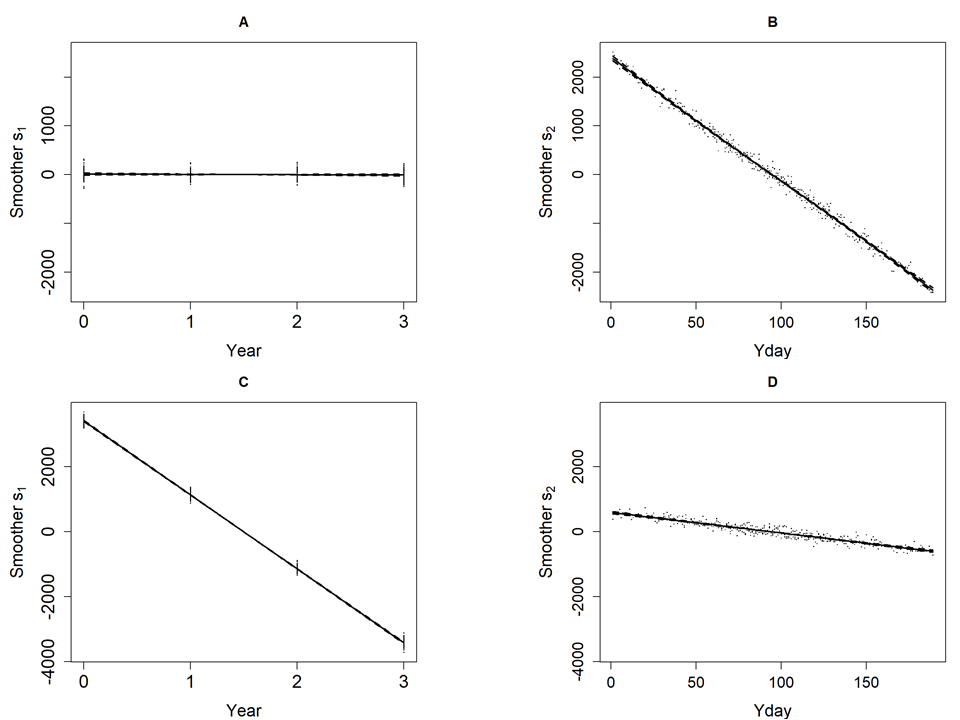
**

**S5 Figure 5.** Smoothers (solid) s1 for Year and s2 for Yday, with 95% confidence bands (dashed) and partial residuals (dots) obtained by fitting the GAM model to the Low Variation *incomplete data* of (A-B) Scenario 1 and bottom row (C-D) Scenario 2. For Scenario 1, these respectively indicate (A) no statistically significant long-term trend (p=0.339); (B) the seasonal decline pattern is highly statistically significant (p= 2e-16). Conversely, for Scenario 2, (C) the long-term downward trend is highly statistically significant (p= 2e-16); (D) the seasonal linear downward trend is highly statistically significant (p= 2e-16). The fitted GAM model diagnostics were reasonable for both Scenarios (see S4 Figures (8-9).

**
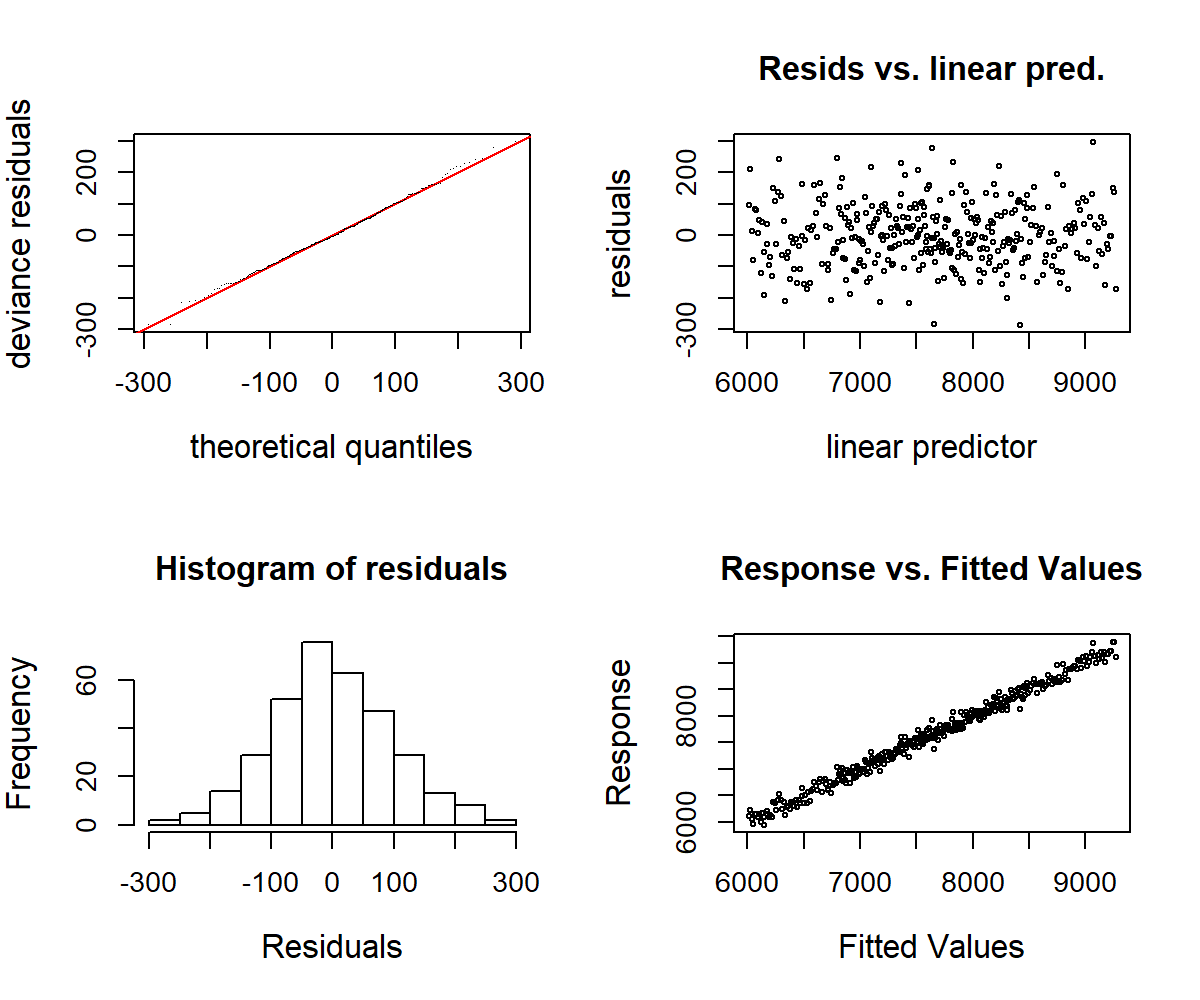
**

**S5 Figure 6.** Diagnostics plots for the GAM model shown in S5 Figure 4(AB) fitted to the Scenario 1 Low Variation *paired data*. The left column (top) shows a qq plot with (bottom) histogram to assess normality, the right column (top) shows residuals v fitted values to assess homogeneity, with (bottom) response v fitted values.

**
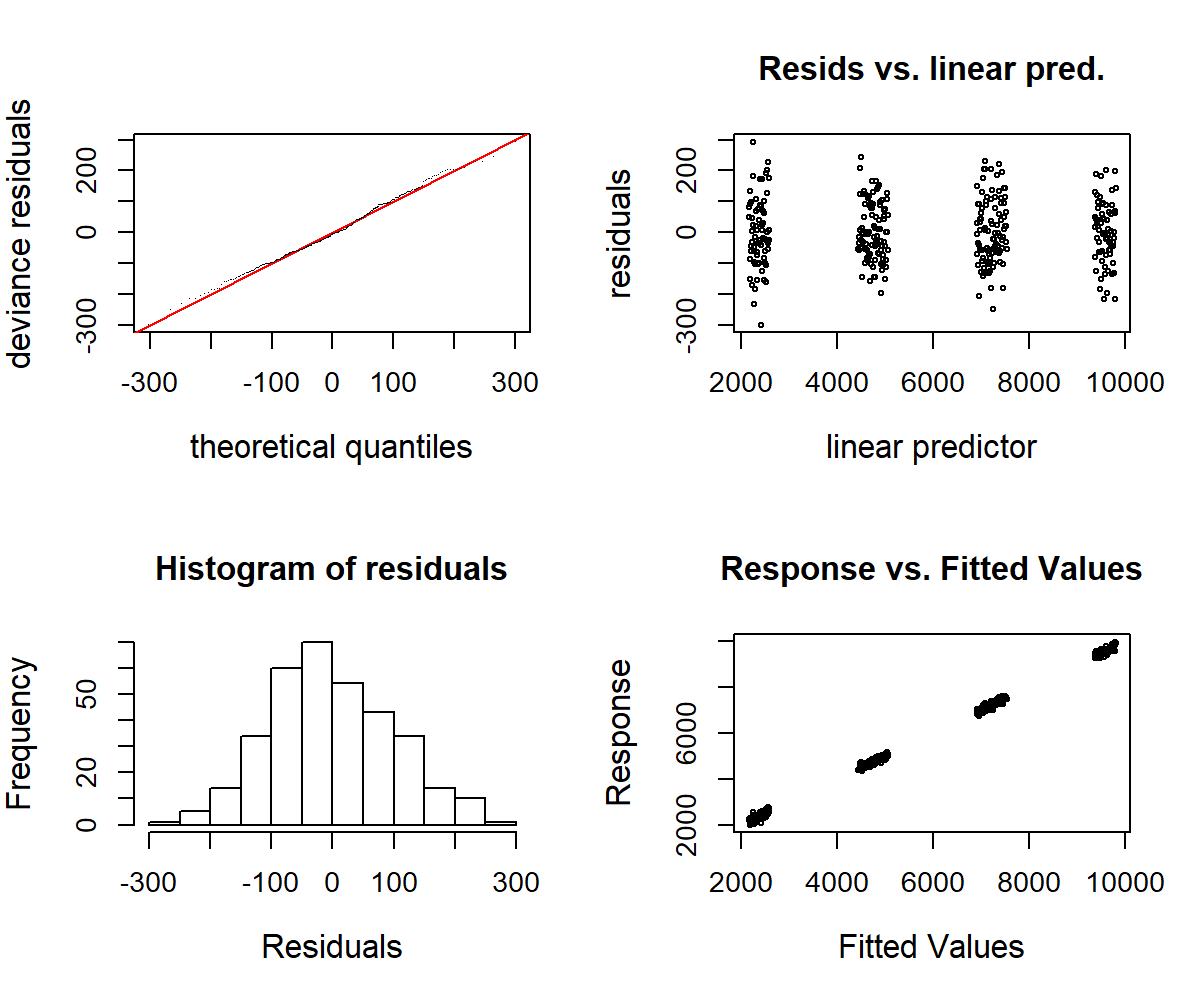
**

**S5 Figure 7.** Diagnostics plots for the GAM model of S5 Figure 4(CD) fitted to the Scenario 2 low Variation *paired data*. The left column (top) shows a qq plot with (bottom) histogram to assess normality, the right column (top) shows residuals v fitted values to assess homogeneity, with (bottom) response v fitted values.

**
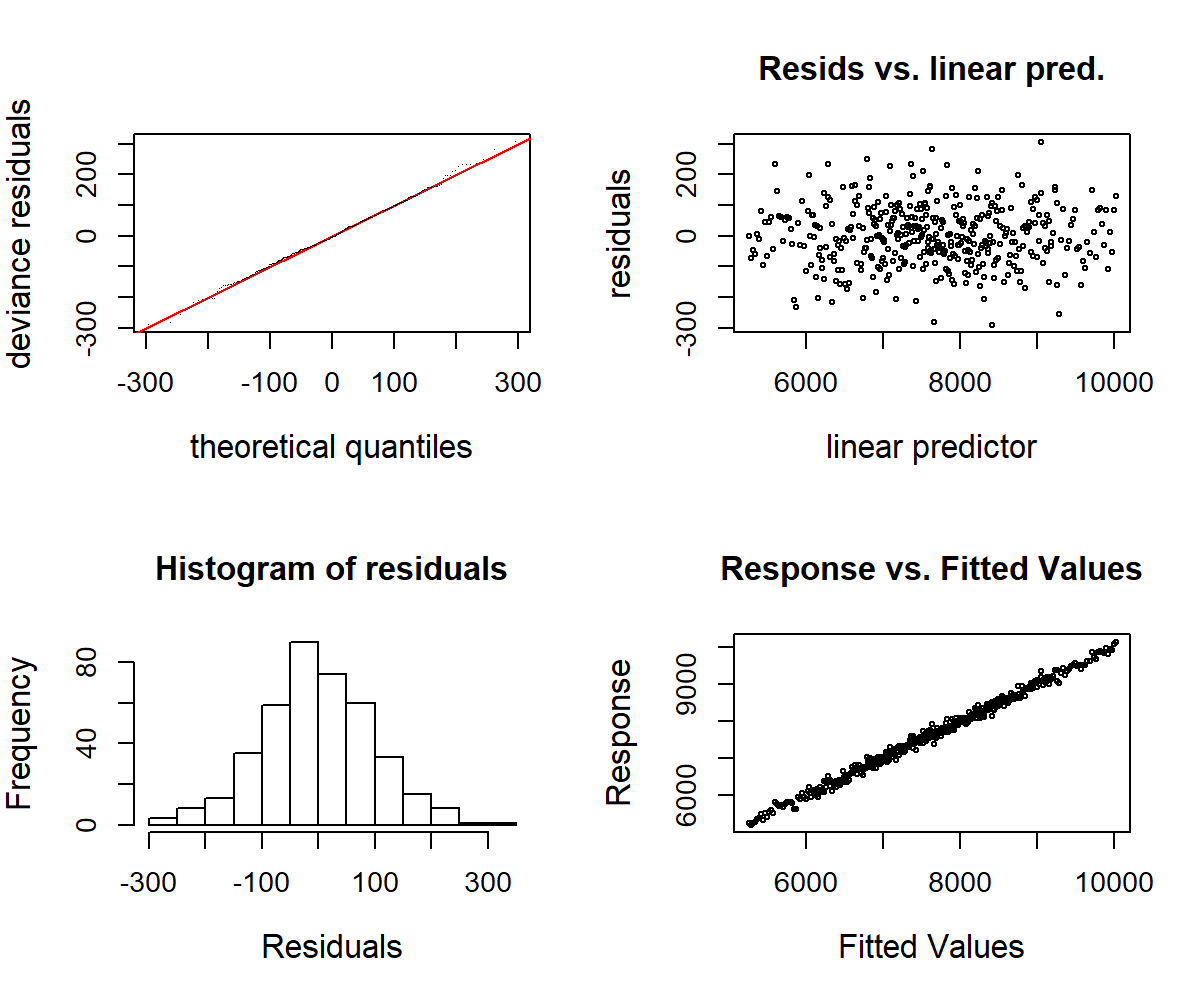
**

**S5 Figure 8.** Diagnostics plots for the GAM model of S5 Figure 5(AB) fitted to the of Scenario 1 Low Variation *incomplete data*. The left column (top) shows a qq plot with (bottom) histogram to assess normality, the right column (top) shows residuals v fitted values to assess homogeneity, with (bottom) response v fitted values.

**
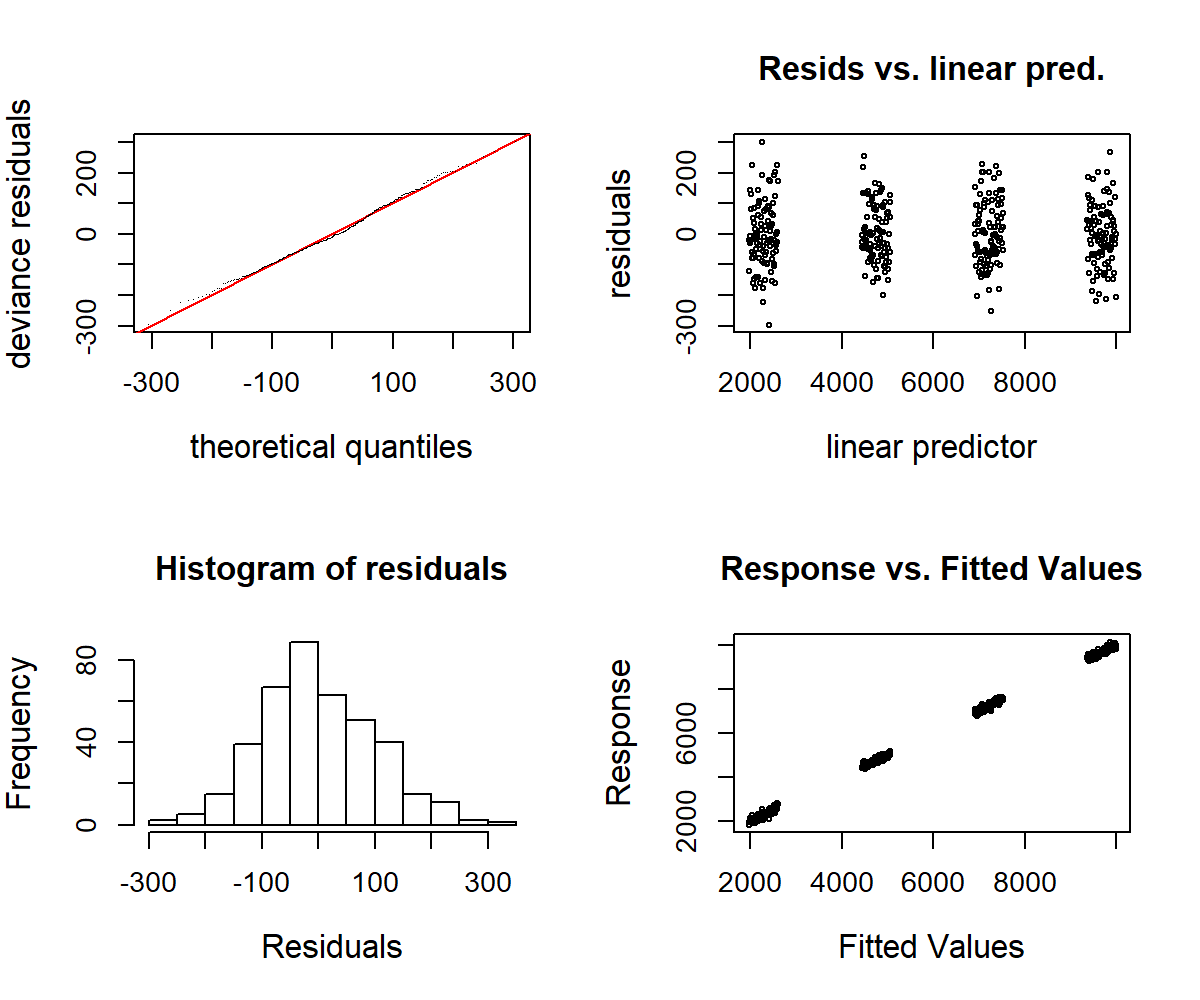
**

**S5 Figure 9.** Diagnostics plots for the GAM model of S5 Figure 5(CD) fitted to the Scenario 2 Low Variation *incomplete data* . The left column (top) shows a qq plot with (bottom) histogram to assess normality, the right column (top) shows residuals v fitted values to assess homogeneity, with (bottom) response v fitted values.
