# Supplementary material for: Estimating cetacean population trends from static acoustic monitoring data using Paired Year Ratio Assessment (PYRA)
Source: PLoS One. 2022 Mar 17;17(3):e0264289. doi: 10.1371/journal.pone.0264289 (PMC8929582; doi:10.1371/journal.pone.0264289)
Supplement: S1 Table — Comparison of PYRA with the four randomisation trend tests applied to the synthetic data of (S6 Table 1 in S1 Table) Scenario 1 and (S6 Table 2 in S1 Table) Scenario 2 either with High or Low variation. The sample statistic Stat was determined by the randomisation test and is shown with p-values for each data set. RT1: Linear regression with the regression slope coefficient m as the sample statistic; RT2: Number of runs above or below the median value of the data; RT3: Number of runs of positive or negative differences; RT4: Count of positive or negative differences between consecutive data points. The PYRA trend statistic PT^ with 95% percentile confidence limits (lower, upper). 5000 randomisations were used to generate the randomisation distributions and PYRA percentile confidence intervals throughout. (DOCX) [file pone.0264289.s004.docx]

**S6 Tables 1 and 2. PYRA and Randomisation tests for Synthetic High and Low Variation data.** Comparison of PYRA with the four randomisation trend tests applied to the synthetic data of (S6 Table 1) Scenario 1 and (S6 Table 2) Scenario 2 either with High or Low variation. The sample statistic *Stat* was determined by the randomisation test and is shown with p-values for each data set. RT1: Linear regression with the regression slope coefficient *m* as the sample statistic; RT2: Number of runs above or below the median value of the data; RT3: Number of runs of positive or negative differences; RT4: Count of positive or negative differences between consecutive data points. The PYRA trend statistic $\hat{P_{T}}$ with 95% percentile confidence limits (lower, upper). 5000 randomisations were used to generate the randomisation distributions and PYRA percentile confidence intervals throughout.

| **S6 Table 1** | RT1 | | RT2 | | RT3 | | RT4 | | PYRA |
| --- | --- | --- | --- | --- | --- | --- | --- | --- | --- |
| **Scenario 1 data** | Stat *m* | p value | Stat | p value | Stat | p value | Stat | p value | $\hat{P_{T}}$ |
| *High variation* |  |  |  |  |  |  |  |  |  |
| Complete | -1.6614 | < 0.0001 | 210 | < 0.0001 | 965 | 0.3138 | 707 | 0.0262 | N/A |
| Incomplete | -2.1351 | < 0.0001 | 134 | < 0.0001 | 261 | 0.2758 | 198 | 0.4444 | N/A |
| Paired | -1.4992 | < 0.0001 | 133 | < 0.0001 | 218 | 0.1526 | 169 | 0.4972 | 0.995 (0.932-1.003) |
| *Low variation* |  |  |  |  |  |  |  |  |  |
| Complete | -1.56 | < 0.0001 | 28 | < 0.0001 | 937 | 0.0126 | 617 | < 0.0001 | N/A |
| Incomplete | -1.9879 | < 0.0001 | 181 | < 0.0001 | 254 | 0.0782 | 163 | < 0.0001 | N/A |
| Paired | -1.4644 | < 0.0001 | 18 | < 0.0001 | 218 | 0.1674 | 138 | < 0.0001 | 0.999 (0.941-1.005) |

| **S6 Table 2** | RT1 | | RT2 | | RT3 | | RT4 | | PYRA |
| --- | --- | --- | --- | --- | --- | --- | --- | --- | --- |
| **Scenario 2 data** | Stat *m* | p value | Stat | p value | Stat | p value | Stat | p value | $\hat{P_{T}}$ |
| *High variation* |  |  |  |  |  |  |  |  |  |
| Complete | -6.243 | < 0.0001 | 158 | < 0.0001 | 955 | 0.1336 | 724 | 0.3076 | N/A |
| Incomplete | -6.3579 | < 0.0001 | 44 | < 0.0001 | 261 | 0.2866 | 199 | 0.4840 | N/A |
| Paired | -6.3988 | < 0.0001 | 44 | < 0.0001 | 218 | 0.1592 | 169 | 0.5086 | 0.666 (0.629-0.688) |
| *Low variation* |  |  |  |  |  |  |  |  |  |
| Complete | -6.2367 | < 0.0001 | 22 | < 0.0001 | 970 | 0.4358 | 705 | < 0.0001 | N/A |
| Incomplete | -6.239 | < 0.0001 | 2 | < 0.0001 | 254 | 0.0816 | 191 | 0.0822 | N/A |
| Paired | -6.2325 | < 0.0001 | 2 | < 0.0001 | 215 | 0.0762 | 162 | 0.1012 | 0.680 (0.652-0.699) |
