# Supplementary material for: Estimating cetacean population trends from static acoustic monitoring data using Paired Year Ratio Assessment (PYRA)
Source: PLoS One. 2022 Mar 17;17(3):e0264289. doi: 10.1371/journal.pone.0264289 (PMC8929582; doi:10.1371/journal.pone.0264289)
Supplement: S1 Appendix — (DOCX) [file pone.0264289.s005.docx]

**S1 Appendix. Calculation of Regional PYRA from multiple acoustic site data.**

For a region R in which data are collected from a number of sites J, a regional PYRA may be represented by $\hat{P}_{R}\left( t_{k}^{*} \right)$ calculated by combining the paired data $Y_{1}\left\{ \left( t_{kj}^{*},y_{kj}^{*} \right) \right\}$ and $Y_{2}\left\{ \left( t_{kj+C}^{*},y_{kj+C}^{*} \right) \right\}$ recorded at the separate sites j= 1 to J. Because gaps in the separate site data time series will typically not all correspond in time, there are two main options for combining the paired data across the sites to produce a regional average.

Either:

1. Sum the site-paired data sets $Y_{1}\left\{ y_{kj}^{*} \right\}$ and $Y_{2}\left\{ y_{kj+C}^{*} \right\}$ across all the sites j =1 to J to respectively obtain

$$Y_{k}^{*}=\sum_{j=1}^{J} y_{\mathrm{kj}}^{*} (A1)$$

and

$$Y_{(k+C)}^{*}=\sum_{j=1}^{J} y_{kj+C}^{*} (A2)$$

so that

$$\hat{P}_{R}\left( t_{k}^{*} \right)=\frac{m_{I}\left( Y_{(t+C)}^{*} \right)}{m_{I}\left( Y_{t}^{*} \right)} (A3)$$

or

1. Calculate the separate site $\hat{P}_{j}\left( t_{k}^{*} \right)$ from the paired data for each site j=1 to J sites and then take the weighted average so that

$$\hat{P}_{R}\left( t_{k}^{*} \right)=\sum_{j=1}^{J} w_{j}\hat{P}_{j}\left( t_{k}^{*} \right) (A4)$$

where w_j_ is an appropriate weighting, such as the proportion of the total clicks received at each site.

The alternative calculation (2) would be more appropriately applied to situations where information on any site-specific trends is likely to be seen as important in a population abundance assessment. In this case, caution is required to ensure that the weightings ( or lack of weightings) assigned to site ratios does not disproportionality influence the regional evaluation of $\hat{P}_{R}\left( t_{k}^{*} \right)$ by invoking Simpson’s Paradox.
