# Supplementary material for: Estimating cetacean population trends from static acoustic monitoring data using Paired Year Ratio Assessment (PYRA)
Source: PLoS One. 2022 Mar 17;17(3):e0264289. doi: 10.1371/journal.pone.0264289 (PMC8929582; doi:10.1371/journal.pone.0264289)
Supplement: S2 Appendix — S2 Figure 1 shows smoothers (solid line); s1 for year and s2 for day within the year (Yday), with 95% confidence bands (dashed) and partial residuals (dots) obtained by fitting the GAM model to the incomplete data of (A-B) Scenario 1 and bottom row (C-D) Scenario 2. (DOCX) [file pone.0264289.s006.docx]

**S2 Appendix. GAMs plots for the Synthetic *incomplete data* of Scenario1 and Scenario 2 .** S2 Figure 1 shows Smoothers (solid line); s_1_ for year and s_2_ for day within the year (Yday), with 95% confidence bands (dashed) and partial residuals (dots) obtained by fitting the GAM model to the *incomplete data* of (A-B) Scenario 1 and bottom row (C-D) Scenario 2.


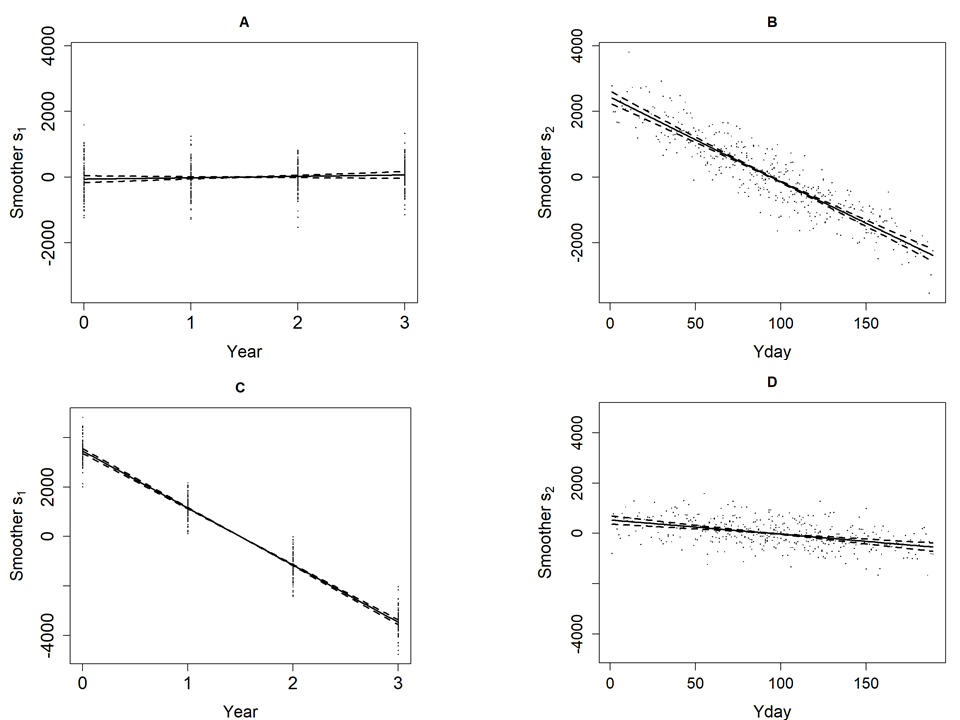


**S2 Figure 1**
